# An edge-based approach for virtual network embedding based on the graph edit distance

**DOI:** 10.1371/journal.pone.0288037

**Published:** 2023-07-07

**Authors:** Zexi Xu, Lei Zhuang, Mengyang He, Sijin Yang, Yu Song, Junli Guo, Wencui Li

**Affiliations:** 1 The School of Computer and Artificial Intelligence, Zhengzhou University, Zhengzhou, Henan, China; 2 The School of Cyber Science and Engineering, Zhengzhou University, Zhengzhou, Henan, China; 3 Song Shan Laboratory, Zhengzhou, Henan, China; 4 The School of Softeware, Zhengzhou University, Zhengzhou, Henan, China; 5 The State Grid Henan Information & Telecommunication Company (Data Center), Zhengzhou, Henan, China; TU Wien: Technische Universitat Wien, AUSTRIA

## Abstract

Virtualization and resource isolation techniques have enabled the efficient sharing of networked resources. How to control network resource allocation accurately and flexibly has gradually become a research hotspot due to the growth in user demands. Therefore, this paper presents a new edge-based virtual network embedding approach to studying this problem that employs a graph edit distance method to accurately control resource usage. In particular, to manage network resources efficiently, we restrict the use conditions of network resources and restrict the structure based on common substructure isomorphism and an improved spider monkey optimization algorithm is employed to prune redundant information from the substrate network. Experimental results showed that the proposed method achieves better performance than existing algorithms in terms of resource management capacity, including energy savings and the revenue-cost ratio.

## Introduction

Network virtualization [1] is an integral component of current and future networks and promotes the development of networked technologies such as cloud computing [2]. Due to virtualization, network operators have opportunities to consolidate their equipment into standardized high-volume components. This is reflected by efficiently utilizing substrate network (SN) resources through sharing among several virtual networks requests (VNRs) [3]. Virtualization introduces great flexibility in terms of “where” VNRs can be embedded. However, exploiting this resource allocation problem poses a fundamental algorithmic challenge.

**Fig 1:**
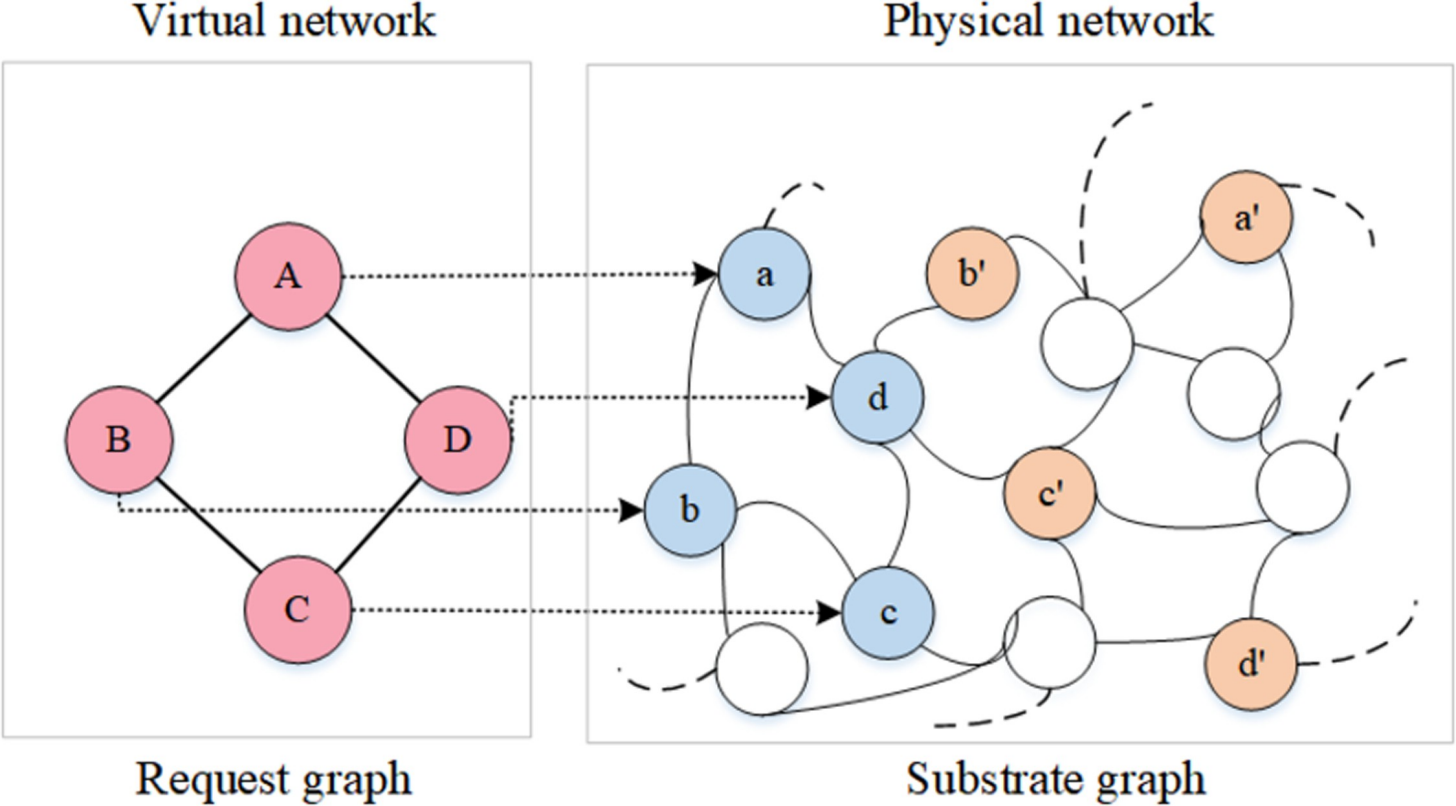
An example of embedding a virtual network request on the physical substrate network, where the virtual network (left) is presented as a request graph describing the resource requirements both on nodes and links; substrate network (right) is presented as a substrate graph describing the physical infrastructure and its resources.

In general, the resource allocation problem is known as the virtual network embedding (VNE) problem [4], which can be posed as a graph-theoretic problem: both VNR, describing a workload, and SN, describing the physical infrastructure, can be modeled as graphs. To better explain the VNE problem, Fig 1 shows a physical network of numerous nodes and edges receiving a VNR with four virtual nodes and four virtual links. To realize the embedding of this request, the VNE algorithm will search for candidate physical resources in the substrate network graph that have enough residual capacities to host its demands, and Fig 1 shows two embedding solutions for this VNR, identified in blue and yellow, respectively. In solution 1 (blue), virtual nodes A, B, C and D are hosted on substrate nodes a, b, c and d, and the virtual links A-B, A-D, B-C and C-D are subsequently hosted on substrate links a-b, a-d, b-d and c-d. The same procedure repeats in solution 2 (yellow), where virtual nodes A, B, C and D will be embedded on substrate nodes a’, b’, c’ and d’. Although both solutions are feasible, it is not difficult to find the difference between them: solution 2 opens up more link resources than solution 1, which means that solution 2 takes up more redundant resources and more latency than solution 1. Therefore, a VNE algorithm with strong resource management capabilities needs to search for candidate physical nodes that can satisfy the demands while maintaining similar connectivity to virtual requests.

However, this feature has been largely overlooked in previous studies [5–7]. Most related works are "straightforward" in solving the VNE problem, which focusing only on resource availability [8–12] to find the most adequately resourced physical nodes in the entire physical network space to host virtual nodes. As applications of VNE range from mapping testbeds over embedding batch-processing tasks to embedding service function chains with different embedding restrictions on nodes and edges, not only capacity [13] but also latency [14] and power consumption [15], such a strategy that ignores the relationship between demands (i.e., VN) and services (i.e., SN) may face a dilemma: nodes selected without structure restriction may have a scattered location, which results in the use of more substrate resources. With the physical nodes powered up and links segmented, would most probably result on rejecting some virtual demands, or including additional hidden SN resources, accordingly, increasing embedding costs and consuming more energy.

Therefore, more and more research performs virtual nodes mapping and virtual links mapping no longer as two separate subproblems but with some coordination between their solutions, and according to predefined constraints, performing resource allocation by building a substrate resource management region [16–18]. By using a feedback loop, spectral clustering and other methods, several nodes and links in the substrate graph are formulated into a collection, which is used to allocate all or part of the networked resource requirements in the VNR. However, although these approaches provide some limitations on the location of node mapping, they have two drawbacks that cannot be ignored: (1) the impact of structural similarity between requests and services on the final embedding result is not fully considered. In fact, according to Fig 1, when realizing the embedding for VNR demands, an efficient VNE algorithm needs not only to find a physical node for each virtual node that meets the demands, but also to maintain the similar connections as the request, as a similar structure allows open fewer physical link resources and the more similar the hosted physical graph is to the request graph, the fewer redundant resources are opened. (2) The impact of search space on the final embedding result is ignored. In order to avoid inefficient usage due to under-exploitation of physical resources in the substrate network graph, full-scale search in the underlying network is required. However, the information of the substrate graph is not always useful [19], even some information is redundant. To quickly find the most accurate substrate resource management region, a fast and efficient optimization method [20] is needed to help find the most potential physical resources.

Consequently, in this paper, by finding the most potential substrate resource management region, we propose an edge-based VNE algorithm, abbreviate as VNE_MR for providing predictable performance guarantees on all resources. Specifically, different from previous algorithms that focus on the nodes, we abstract the virtual network as a request graph, denoted by *G*_*v*_, and employ an edge-based graph edit distance method CSI_GED [21] following the links to edit *G*_*v*_ to the physical resource management region with a similar size to *G*_*v*_, denoted by *R*(*G*_*v*_). In addition, to control the hosted physical network structure efficiently and support various requirements flexibly, we restrict the use conditions of network resources and restrict the structure of *R*(*G*_*v*_) based on common substructure isomorphism. Nevertheless, to find the highest potential *R*(*G*_*v*_) to host the *G*_*v*_ as the VNE solution, a modified spider monkey optimization (SMO) [22] algorithm is designed to prune redundant information from the substrate network and to find the highest potential *R*(*G*_*v*_) in a parallel manner. So that after only a few iterations, the candidate resources are delimited in a region similar to the size of the request graph, which not only keeps the full exploration of the substrate network but also controls the cost of optimization in an small value.

Main contributions:

1. To perform resource allocation according to predefined constraints, we model the VNE problem using graph theory and transform it into the problem of building a substrate resource management region based on an edge-based graph editing distance method.
2. To find the VNE solution with low cost, a modified SMO optimization algorithm is designed to prune redundant information from the substrate network and to find the highest potential substrate resource management region in a parallel manner.
3. Experimental results showed that the proposed method achieves better performance than existing online VNE algorithms in terms of resource management capacity, including energy savings and the revenue-cost ratio.

The remainder of the paper is organized as follows. Section 2 reviews related studies. Embedding preliminaries are defined in Section 3. Section 4 introduces the proposed VNE_MR algorithm in detail. An explanation of the proposed algorithm and a discussion of its results are presented in Section 5. Finally, Section 6 concludes the paper and highlights certain future work.

## Related work

In existing VNE research, a common method focuses on resource availability when addressing the resource allocation problem and managing substrate resources.With regard to resource availability, the most referenced approach is that suggested by Chowdhury et al.[10], which embeds virtual nodes onto SN nodes based on their residual capacities and coordinates edge embedding using the multicommodity flow algorithm. In addition, with the increase in delay-sensitive services [23,24] on the network, a smaller and guaranteed transmission delay is required. Therefore, researchers are starting to consider delay as an important factor when considering the VNE problem. Based on the Google PageRank algorithm and considering link propagation delay as a node-link constraint in the VNE problem, Cao et al. [11] designed a VNE algorithm, denoted as VNE-TAGRD, to manage the underlying network resources. And Shi et al. [12] also based on this devised a node-ranking approach for SDN-based virtual network embedding. However these resource availability-based algorithms ignored the relationship between requests and services. Nodes selected in this manner may be scattered, longer paths could be used, which would result on adding additional costs.

Therefore, some researchers pay more attention on allocating resources according to predefined constraints by building a resource management unit. For example, to manage resources, Khaled et al. [16] proposed a resource set format, called segments, to manage and match resources in segments. They modeled the VNE problem as a two-stage mapping problem and introduced a VNE algorithm called OPaCoVNE to solve the resource management problem while considering the end-to-end delay as the embedded constraint. However, due to the under-exploitation of physical resources in the substrate network, its resource utilization efficiency is low. He et al. [17] used spectral clustering based on field theory to extract substrate network features and manage physical resources. Then, they developed dynamic regions of interest to find embedding areas with energy-saving potential for virtual networks. However, they did not consider the effect of the similarity between the request graph and the service graph on the final embedding result, so some unnecessary costs may be introduced.

In addition, some researchers have proposed some effective VNE methods based on heuristic methods. For example, aiming at maximizing the revenues by mapping more virtual nodes and minimizing the energy cost, Zhang et al. [25] leveraged niche particle swarm optimization technique to design a meta-heuristic algorithm to solve the VNE problem. To manage substrate resources, Jahani et al. [26] designed a D-reachability algorithm to assign reachability rank to resources, modeled VNE as a multiobjective optimization problem, and pursued a nondominated sorting heuristic genetic algorithm to solve it. However, to the best of our knowledge current heuristics are combined with the resource availability and are used to fully explore the substrate network resources. Thus they are still essentially resource availability-based approaches, which aim at finding the most sufficient resources. This implies that the search space in finding candidate physical nodes for each node is the entire underlying network, and thus more iterations are often required in exploring the substrate network to obtain the optimal embedding solution, which resulting in a high cost.

## Preliminaries

In this section, VN and SN are modeled as the request graph and the substrate graph, respectively. Some propositions about resources, i.e., nodes and links in the VNE problem, are reviewed (Propositions 1–7). In addition, Proposition 8, presenting the common substructure isomorphism, is specifically proposed for our VNE_MR algorithm.

### Substrate network model

We model the substrate network as an undirected graph *G*_*s*_ *=* (*N*_*s*_,*L*_*s*_). This paper also refers to it as a substrate graph, where *N*_*s*_ is the set of all substrate nodes. Each substrate node *m*∈*N*_*s*_ is characterized by its node capacity-available CPU, denoted as *C*(*m*). With respect to substrate links, we consider any link as a pair of nodes, and each substrate link *l*_*mn*_ has a finite bandwidth *B*(*l*_*mn*_) and a substrate link propagation delay *D*(*l*_*mn*_).

### VN request model

In the VNE research area, each VN can also be modeled as a weighted graph *G*_*v*_ = (*N*_*V*_, *L*_*V*_). This paper also refers to it as a request graph, where *N*_*V*_ is the set of all virtual nodes and *L*_*v*_ is the set of all virtual links. Each virtual node *M*∈*N*_*V*_ is characterized by the required CPU, denoted as *C*(*M*). With respect to virtual links, each virtual link *L*_*MN*_ has a required bandwidth *B*(*L*_*MN*_) and required virtual link propagation delay *D*(*L*_*MN*_). By adding the time attributes (e.g., maximum waiting time, arrival time, duration time, leaving time), the VN is extended to a VNR.

### VNE propositions

#### • Proposition 1: node mapping


$$\forall M\in G_{v},m\in G_{s}:x_{M}^{m}=\left\{\begin{array}{l} 1,\;\text{if}\;M\;\text{is}\;\text{mapped}\;\text{on}\;m\\ 0,\;\text{otherwise}\; \end{array}\right.$$ (1)

In (1), if $x_{M}^{m}=1$, virtual node M is mapped to physical node m.


$$\forall M\in G_{v}:\sum_{M\in G_{v}}x_{m}^{M}=1$$ (2)

$$\forall m\in G_{s}:\sum_{m\in Gs}x_{m}^{M}\leq 1$$ (3)

Eqs (2) and (3) ensure that a virtual node must correlate with just one substrate node.

#### • Proposition 2: node capacity


$$\forall M\in G_{v},\forall m\in G_{s}:x_{M}^{m}C(M)\leq C(m)$$ (4)

As shown in (4), to successfully embed a VN, all the virtual nodes must be embedded on substrate nodes with adequate capacity.

#### • Proposition 3: link mapping


$$\begin{array}{l} \forall L_{MN}\in G_{v},\;\forall l_{mn}\in G_{s}:\\ x_{L_{MN}}^{l_{mn}}=\left\{\begin{array}{l} L_{MN},\;\text{if}\;L_{MN}\;\text{is}\;\text{mapped}\;\text{on}\;l_{mn}\\ 0,\;\text{otherwise}\; \end{array}\right. \end{array}$$ (5)

In (5), if $x_{L_{MN}}^{l_{mn}}=1$, virtual link *L*_*MN*_ is mapped on physical link *l*_*mn*_.


$$\begin{array}{l} \forall L_{MN}\in G_{v},\;\forall l_{mn}\in G_{s}:\\ \sum_{l_{mn}}x_{L_{MN}}^{l_{mn}}-\sum_{L_{MN}}x_{L_{MN}}^{l_{mn}}=\left\{\begin{array}{l} 1,\;\text{if}\;x_{M}^{m}=1\\ \text{n},\;\text{if}\;x_{N}^{m}=1\\ 0,\;\text{otherwise}\; \end{array}\right. \end{array}$$ (6)

Eq (6) specifies that the physical path can be split.

#### • Proposition 4: link bandwidth and delay

$$\begin{array}{l} \forall L_{MN}\in G_{v},\forall l_{mn}\in G_{s}:\\ x_{L_{MN}}^{l_{mn}}\cdot B\left(L_{MN}\right)\leq B\left(l_{mn}\right)\;\text{and}\;m\neq n \end{array}$$ (7)

$$\begin{array}{l} \forall L_{MN}\in G_{v},\forall l_{mn}\in G_{s}:\\ x_{L_{MN}}^{l_{mn}}\cdot D\left(L_{MN}\right)\leq D\left(l_{mn}\right)\;\text{and}\;m\neq n \end{array}$$ (8)

As shown in (7) and (8), to successfully embed *G*_*v*_, all the virtual links must be embedded on substrate links with available bandwidth and delay.

#### • Proposition 5: node energy consumption

$$\begin{array}{l} \forall m\in G_{s},\\ E_{m}=\left\{\begin{array}{l} m._{base}+pl*\tau \\ \;\text{if}\;\text{the}\;\text{selected}\;\text{physical}\;\text{node}\;\text{n}\;\text{is}\;\text{open}\;\\ 0,\;\text{otherwise}\; \end{array}\right. \end{array}$$ (9)

where *m*._*base*_ is the baseline power without any central processing unit (CPU) load, *pl* represents the energy proportion factor, *m*._*max*_ denotes the total power at maximum capacity (*pl* = *m*._*max*_*—m*._*base*_), and τ denotes the CPU utilization of node m (*τ* = *C*(*M*)/*C*(*m*)). When the node is powered off or in the hibernation state, the energy consumption of the node is 0.

#### • Proposition 6: link energy consumption

$$\begin{array}{l} \forall l_{mn}\in G_{s},\\ E_{m}=\left\{\begin{array}{l} l_{mn.base,}\\ \;\text{if}\;\text{the}\;\text{selected}\;\text{physical}\;\text{link}\;\text{n}\;\text{is}\;\text{open}\;\\ 0,\;\text{otherwise}\; \end{array}\right. \end{array}$$ (10)

*l*_*mn*_._*base*_ indicates the link energy consumption, which is generally constant. When the link is powered off or in the hibernation state, the link energy consumption s 0.

#### • Proposition 7: the status of substrate resources

The variable *s*^*t*^ is a binary variable that demonstrates that the substrate node or link is turned on/off at time *t* and is described in (11). It should be noted that the turned-on (not-off) servers should have a greater chance of mapping because they need less electrical energy to host new nodes.


$$s^{t}=\left\{\begin{array}{l} 1,\;\text{if}\;\text{the}\;\text{physical}\;\text{resource}\;\text{is}\;\text{turned}\;\text{on}\;\text{at}\;\text{time}\;t\\ 0,\;\text{if}\;\text{the}\;\text{physical}\;\text{resource}\;\text{is}\;\text{turned}\;\text{off}\;\text{at}\;\text{time}\;t \end{array}\right.$$ (11)

As shown in (11), if $s^{t}=1$, the status of the substrate node or link is turned on.

#### • Proposition 8: common substructure isomorphism

Given two graphs *G*_1_ = (*N*_1_, *L*_1_) and *G*_*2*_ = (*n*_2_, *l*_*2*_), if ∃*f*: *N*_1_→*n*_2_. Then, we must be able to find link *L*_1_→*l*’ among them induced by *f*, where $L_{1}={\text{U}}_{(M,N)\in N}\left\{L_{MN}\right\}$, $l'={\text{U}}_{(m,n)\in N'}\left\{L_{mn}\right\}$, where $l^{\prime }\subseteq l_{2}$. The two graphs *G*_1_ = (*N*_1_, *L*_1_) and *G’* = (*n*_2_, *l’*) have the same structure; thus, these nodes and links are a common substructure isomorphism of *G*_1_ and *G*_2_ induced by *f*. As a rule of thumb, the common substructure is typically a minimally connected graph and is not unique in the sense that it can be determined by different maps.

**Fig 2:**
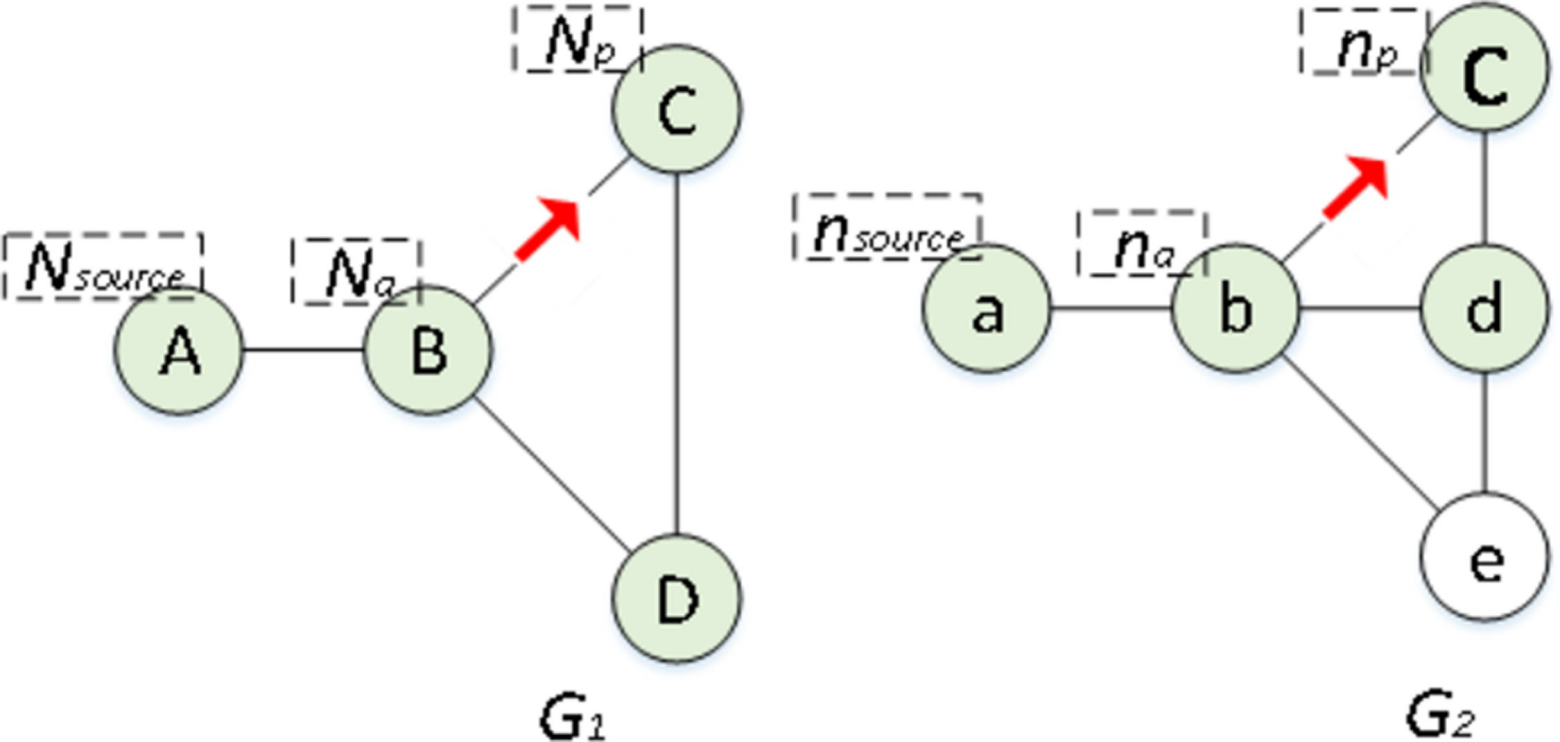
Example of embedding the common substructure isomorphism of *G*_*1*_ (left) to that of *G*_*2*_ (right).

Fig 2 shows the common substructure isomorphism of *G*_*1*_ and *G*_*2*,_ with four nodes colored green, where nodes A and B are mapped to nodes a and b, and edge A-B is mapped to edge a-b. Then, nodes B, C and D need to map to nodes b, c, and d, and edges B-C and B-D need to map to edges b-c and b-d, subsequently following a graph edit function. For this graph edit problem, we set this process beginning with the source node, *N*_*source*_ (the A in *G*_*1*_) and *n*_*source*_ (the a in *G*_*2*_), which are always selected by a given method. Node *N*_*a*_ (B in *G*_*1*_), which has been mapped to *n*_*a*_ (b in *G*_*2*_), is denoted as an active node, and its task is to explore new nodes. Nodes that are surrounded by active nodes are called *passive nodes*, which are denoted as *N*_*p*_ (the C in *G*_*1*_). Its task is to act as the “next” node to be mapped. The nodes in *G*_*2*_ that have not been exploited are called *free nodes*, denoted as *n*_*f*_. The edge in *G*_*1*_ between *N*_*a*_ and *N*_*p*_ is marked as *L*_*ap*_, and the edge in *G*_*2*_ between *n*_*a*_ and *n*_*p*_ is marked as _*lap*._

### Proposed VNE_MR algorithm

In VNE_MR, instead of focusing on research availability and searching the whole substrate network, we narrow the solution space to a certain range by building resource management regions for solving the VNE problem efficiently; however, considering various constraints, the problem is still very complex.

#### Proposition

Building a resource management region with resource constraints is NP-hard.

#### Proof

We use a reduction from the well-known NP-hard problem MAX-SAT [27]. An instance of MAX-SAT is defined by (*CF*,*w*), where *CF* is a set of Boolean clauses such that each clause *C*∈*CF* is a disjunction of literals with a positive weight *w*(*CF*). Let *X* = {*x*_1_,…,*x*_2*n*_} be the set of Boolean variables in the clauses of *CF*. A literal is a variable *x*∈*X* or its negation $\bar{x}$. For each *x*_*i*_∈*X*, let *x*_*i*_ = 1 (*x*_*i*_ = 0 resp.), $x_{n+i}={\bar{x}}_{i}=1-x_{i}$, a clause $C_{j}=x_{j_{1}}\vee x_{j_{2}}\vee \cdots \vee x_{j_{k}}$∈*CF* can be considered a function on *x* as follows:

$$C_{j}=C_{j}(x)=1-\prod_{i=1}^{k_{j}}\left(1-x_{j_{k}}\right)$$ (12)

Thus, its goal, as shown in (13), is to find an assignment of these variables that maximizes the weight of the clauses. The value of a truth assignment *CF* is defined as:

$$\mathrm{Max}\left(\sum_{C_{j}\in CF}w\left(C_{j}\right)C_{j}(x)\right)$$ (13)

If we solve the resource management region, denoted as *R*(*G*_*v*_), and build the problem by considering the resource constraints (Propositions 1 to 7) as variables, taking add one physical resource to *R*(*G*_*v*_) as a clause and taking the energy consumption inverse value as the clause weight, then we can obtain a VNE solution of MAX-SAT: *x*_*1*_ = 1 (or 0) if the capacity of *n*_*a*_ is more than *N*_*a*_; *x*_*2*_ = 1 (or 0) if the capacity of *n*_*p*_ satisfies the requirement of *N*_*p*_; *x*_*3*_ = 1 (or 0) if *n*_*p*_ is turned on; *x*_*4*_ = 1 (or 0) if the bandwidth of *l*_*ap*_ satisfies the constraint of *L*_*ap*_; *x*_*5*_ = 1 (or 0) if the delay of *l*_*ap*_ satisfies the constraint of *L*_*ap*_; *x*_*6*_ = 1 (or 0) if *l*_*ap*_ is turned on. The cost of the solution is equal to the sum of the costs of the clauses, which describes the energy consumption of this *R*(*G*_*v*_). This ends the **proof**.

Therefore, given that building *R*(*G*_*v*_) with resource constraints is an NP-hard problem, we turn our attention to finding a feasible solution that is near optimal by delimiting the highest potential physical region in the substrate network. However, when delimiting *R*(*G*_*v*_), it is not hard to conclude that restrictions in a resource management region that are too tight (in extreme cases, treating each node and link as a unit) will most likely result in rejecting some virtual demands; or restrictions in a resource management region that are too loose (in extreme cases, treating the whole substrate graph as a unit) will occupy many unnecessary physical resources and identify additional hidden nodes, accordingly increasing embedding costs and consuming more power. Therefore, when we delimit *R*(*G*_*v*_), an edge-based graph edit distance method CSI_GED is used to ensure that the size of *R*(*G*_*v*_) is nearly the same as the size of *G*_*v*_. The main advantage in delimiting *R*(*G*_*v*_) relies on its ability to “copy” the information of *G*_*v*_ into *R*(*G*_*v*_) step by step so that *R*(*G*_*v*_) can be controlled at a predicted size. Among this process, we use this approach to edit each element, i.e., virtual nodes and links, in *G*_*v*_ to their counterparts in *R*(*G*_*v*_) and calculate the edit distance from *G*_*v*_ to *R*(*G*_*v*_), considering the following constraints, namely, CPU, bandwidth, and end-to-end delay to find a physical region to host *G*_*v*_.

More details about the steps in our VNE algorithm (VNE_MR) are discussed in the following subsections. The method of delimiting the *R*(*G*_*v*_) in the substrate graph is introduced in Section 4.1, and Section 4.2 introduces an improved SMO algorithm for pruning the substrate graph rapidly to find the highest potential *R*(*G*_*v*_) that is the final near-optimal VNE solution.

### Delimiting the resource management region

In the previous VNE algorithm, the mapping process typically begins with node mapping and then embeds the links by connecting the respective endpoints, which ignores the network topology constraints on mapping location and may scatter the position of the mapped nodes, leading to a high probability that a substrate link may cross several substrate nodes and yield more resources to be occupied.

Therefore, when solving the VNE problem, we propose a method for delimiting *R*(*G*_*v*_) by using an edge-based graph edit distance CSI_GED to edit *G*_*v*_ to *R*(*G*_*v*_), which focuses on links and taking their end nodes follow directly as a byproduct to strictly control the node locations and ensure the coordination between nodes and links. The motivation behind using the link-based approach is to facilitate allocating all virtual resources belonging to a specific VNR on the corresponding substrate resources that have enough resources to host the demands of the virtual nodes and edges without using any additional hidden substrate resources, which guarantees using networked resources accurately.

Before discussing the implementation of this method, we provide a brief introduction to CSI_GED’s basics.

CSI_GED is an edge-based mapping method that is used for computing graph edit distances via common substructure isomorphism enumeration and uses a depth-first backtracking search combined with heuristics to reduce memory requirements and quickly prune a large portion of the mapping search space. The goal is to transform graph *G*_*1*_ into graph *G*_*2*_ (*G*_*1*_ ≠ *G*_*2*_) with minimal cost *c*(*f*), which is defined as:

c(f)=g(f)+h(f) (14)

where *f* is a function of finding the common substructure isomorphism between *G*_*1*_ and G2; *c*(*f*) denotes the total edit cost on the mapped vertices and edges; *g*(*f*) denotes the induced edit cost on the mapped vertices and edges in the common substructure isomorphism; and *h*(*f*) is the edit cost of the remaining part (i.e., remaining nodes and edges in the graph that are not in the common substructure isomorphism).

It is not difficult to find that in this typical graph edit distance computing problem, there are no weights on the nodes or links. However, in the VNE problem, not only are structural constraints embedded in the connection relationship between nodes and links but also resource constraints for them, including capacity, bandwidth and delay, and they are typically nonuniform. Therefore, when editing *G*_*v*_ into *R*(*G*_*v*_), we aim to minimize energy consumption (as shown in Formula 15, where energy consumption is posed as the edit distance), which can best illustrate the cost of this process because every time a node or link is turned on, there’s a corresponding energy consumption, and formulate the bounds for *R*(*G*_*v*_) by presenting the restrictions from two aspects: resource restrictions and structure restrictions. The formulation is given below.

#### Objective:


$$C=\sum_{(m,n,l_{mn})\in R\left(G_{v}\right)}E_{m}+El_{mn}$$ (15)

#### Resources restrictions:

Capacity constraints:

$$\forall M\in G_{v},\;\forall m\in R\left(G_{v}\right):x_{M}^{m}\cdot C(M)\leq C(m)$$ (16)

Bandwidth constraints:

$$\forall L_{MN}\in G_{v},\forall l_{mn}\in R(G_{v}):x_{L_{MN}}^{l_{mn}}\cdot B(L_{MN})\leq B(l_{mn})\;\text{and}\;m\neq n$$ (17)

Delay constraints:

$$\begin{array}{l} \forall L_{MN}\in G_{v},\forall l_{mn}\in R\left(G_{v}\right):\\ x_{L_{MN}}^{l_{mn}}\cdot D\left(L_{MN}\right)\leq D\left(l_{mn}\right)\;\text{and}\;m\neq n \end{array}$$ (18)

Mapping constraints:

$$\begin{array}{l} \forall M\in G_{v},m\;G_{s}:\\ x_{M}^{m}=\left\{\begin{array}{l} 1,\;\text{if}\;M\;\text{is}\;\text{mapped}\;\text{on}\;m\\ 0,\;\text{otherwise}\; \end{array}\right. \end{array}$$ (19)

$$\begin{array}{l} \forall l_{mn}\in G_{s},\\ E_{m}=\left\{\begin{array}{l} l_{mn.base},\\ \;\text{if}\;\text{the}\;\text{selected}\;\text{physical}\;\text{link}\;\text{n}\;\text{is}\;\text{open}\;\\ 0,\;\text{otherwise}\; \end{array}\right. \end{array}$$ (20)

#### Structure restrictions:

Inspired by CSI_GED, we designed the structural restriction of *R*(*G*_*v*_), which is called the common substructure isomorphism constraint:

$$\begin{array}{l} \forall \left(M,N,L_{MN}\right)\in G_{v},\forall \left(m,n,l_{mn}\right)\in R\left(G_{v}\right):\\ \;\text{if}\;x_{L_{MN}}^{l_{mn}}=1;x_{M}^{m}=1 \end{array}$$ (21)

Constraints 16–20 ensure the legal use of resources in *R*(*G*_*v*_); please refer to Section 3.3 for the specific meaning of the symbols. Constraint (21) is inspired by Proposition 8 in Section 3.3, and it ensures that links are allowed to match only if their composing nodes are consistent with the previously matched nodes and the nodes at both ends of the link satisfy the resource constraints. Under the resources and structure restrictions, even though the link map space seems to be relatively large, resource and structure restrictions prune considerable redundant information and sharply reduce the search space.

Since our method is link-based, editing the virtual link set in *G*_*v*_ step by step to a physical link set is our task, and during this process, the parameters of both links must be unanimous (including the end node following directly); additionally, the values also satisfy the demands. Next, we provide more information about the steps for delimiting *R*(*G*_*v*_) by editing *G*_*v*_ into a similar-sized substrate region under the resource and structure restrictions. The pseudocode is shown in Algorithm 1.

**Algorithm 1** delimiting the resource management region

**1:** Input: *G*_*s*_ and *G*_*v*_

**2: While** t≠ 0 **do**

**3:  for** each VNR arriving at the SN randomly at time t

**Formulate** *Set*_*L*_

**4:     for** each *L*_*vw*_ in *Set*_*L*_:

**5:       if** *B*(*l*_*vw*_)>*B*(*L*_*VW*_) and *D*(*l*_*vw*_)<*D*(*L*_*VW*_)

**6:         if** *C*(*n*_*p*_) > *C*(*N*_*p*_) **then** match *L*_*VW*_ to *l*_*vw*_, *w* to *W*

**7:         else** extend to the neighbor node of *w*

**8:       else** split the other link of *V* with sufficient bandwidth, and select the first node whose capacity meets the constraint as *W* from the neighbor node of the node at the other end of the link

**9:**       calculate *g*_*vne*_(*f*) using (15)

**10:**      update the physical resource capacity

**11:**    use **the** shortest path algorithm to match the remaining links

**12:**    calculate *h*_*vne*_(*f*) using (15)

**13:**    record *c*_*vne*_(*f*) and *c*_*vne*_(*f*) = *g*_*vne*_(*f*) + *h*_*vne*_(*f*)

**Fig 3:**
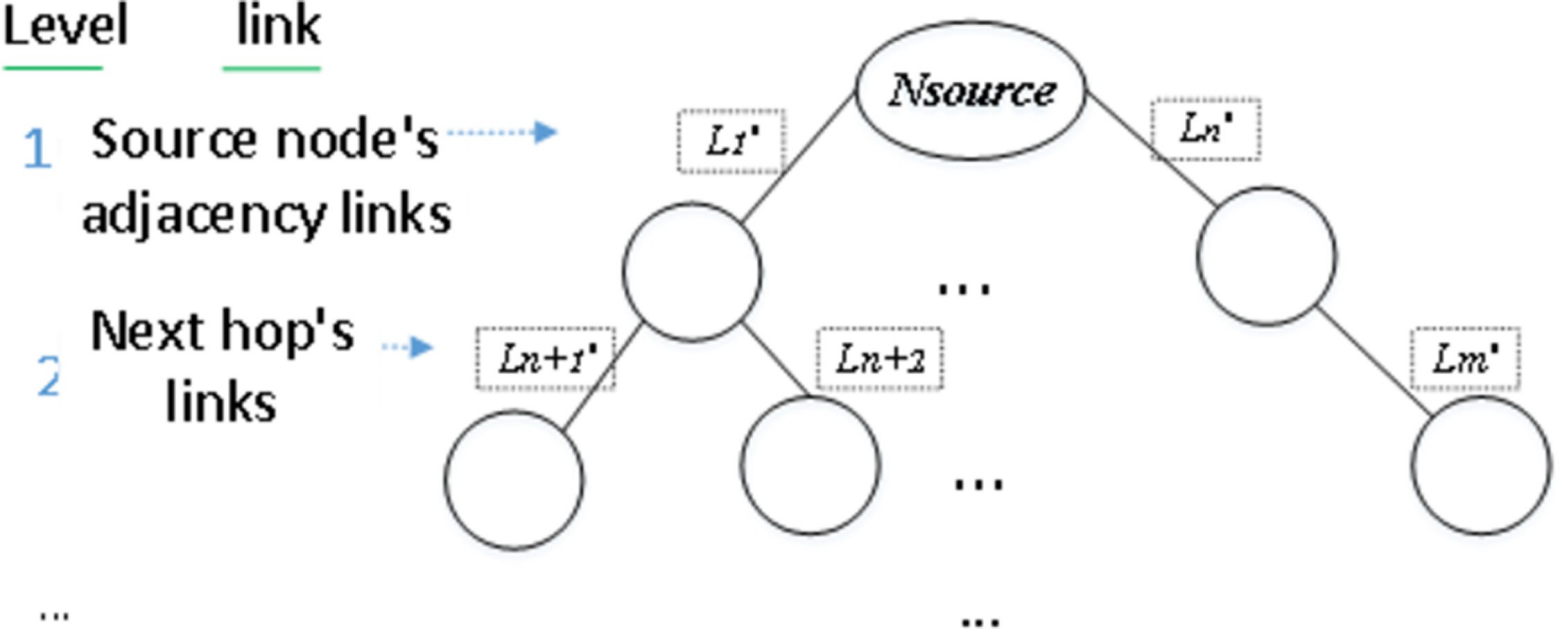
A set of links in a graph starting from *N*_*source*_ rearranged according to bandwidth in a breadth-first manner. The adjacency links of *N*_*source*_ are at the first level, the other links of the adjacency nodes of *N*_*source*_ are at the second level, and so on, until all the links in the whole graph are traversed.

At each time t, if a VNR arrives, first, we need to structure the common substructure isomorphism of *G*_*v*_ and *R*(*G*_*v*_) according to the CSI_GED. For *G*_*v*_, in accordance with the structure restrictions, a minimally connected graph without loops is obtained by using the Prim algorithm (an edge-based minimum spanning tree method), which takes the link bandwidth reciprocal as the weight. Thus, we can obtain a subset of *G*_*v*_ = (*N*, *L*), denoted as *G*_*v*_*’* = (*N*, *L’*), where $L^{\prime }\subseteq L$. Then, we start from *N*_*source*_ and output the link set *Set*_*L*_ = {*L*_*1*_*’*, *L*_*2*_*’* … *L*_*n*_*’*} (where *n*<|*L*|) of *G*_*v*_’ in a breadth-first manner with the link’s bandwidth as the weight (Fig 3). Next, starting from the adjacent link of *n*_*source*_ that is matched to the *N*_*source*_ (introduced in Section 4.2), we search the physical resources that meet the requirements of each link in *Set*_*L*_ in the same breadth-first manner and output the physical resources set *Set*_*l*_ = {*l*_*1*_*’*, *l*_*2*_*’* … *l*_*n*_*’*} corresponding to *Set*_*L*_. In this process, we check links one-to-one directly: if each element in *Set*_*l*_ has enough resources to satisfy the demands of its counterpart in *Set*_*L*_, the first parameter in *G*_*v*_ is compared to the first parameter in *R*(*G*_*v*_), the second parameter to the second, and so on for all the remaining parameters. If the resource constraint check results are true, that is, each virtual link found a matching substrate link, as the nodes follow directly as the byproduct, embedding the virtual nodes and edges is realized together in full coordination onto the corresponding physical substructure isomorphism. Therefore, the resource matching in the common substructure isomorphism can be regarded as a one-stage mapping that highlights the link status. Note that because each virtual node can map to only one physical node, while the links can be split, an energy-saving region must have a compact structure that identifies as few links as possible between a fixed number of physical nodes. However, CSI_GED backtracks the edge mapping space in a depth-first manner, which violates our requirement. Therefore, we modify the search method to proceed in a breadth-first manner.

**Fig 4:**
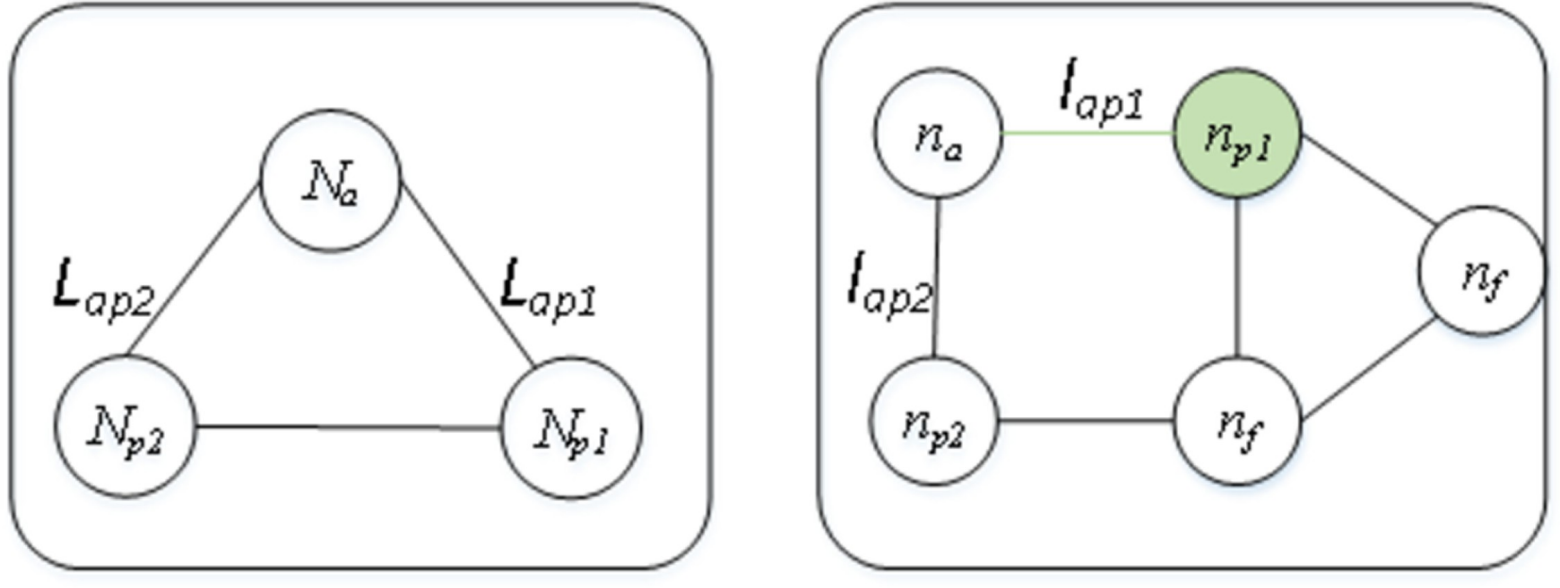
An example of mapping virtual link *L*_*ap1*_ in *G*_*v*_ (left) on physical link *l*_*ap1*_ in *R*(*G*_*v*_) (right) because *l*_*ap1*_ (the green edge) satisfies all the resource constraints, including its byproduct—physical node *n*_*p1*_ (the green node) on the other end.

**Fig 5:**
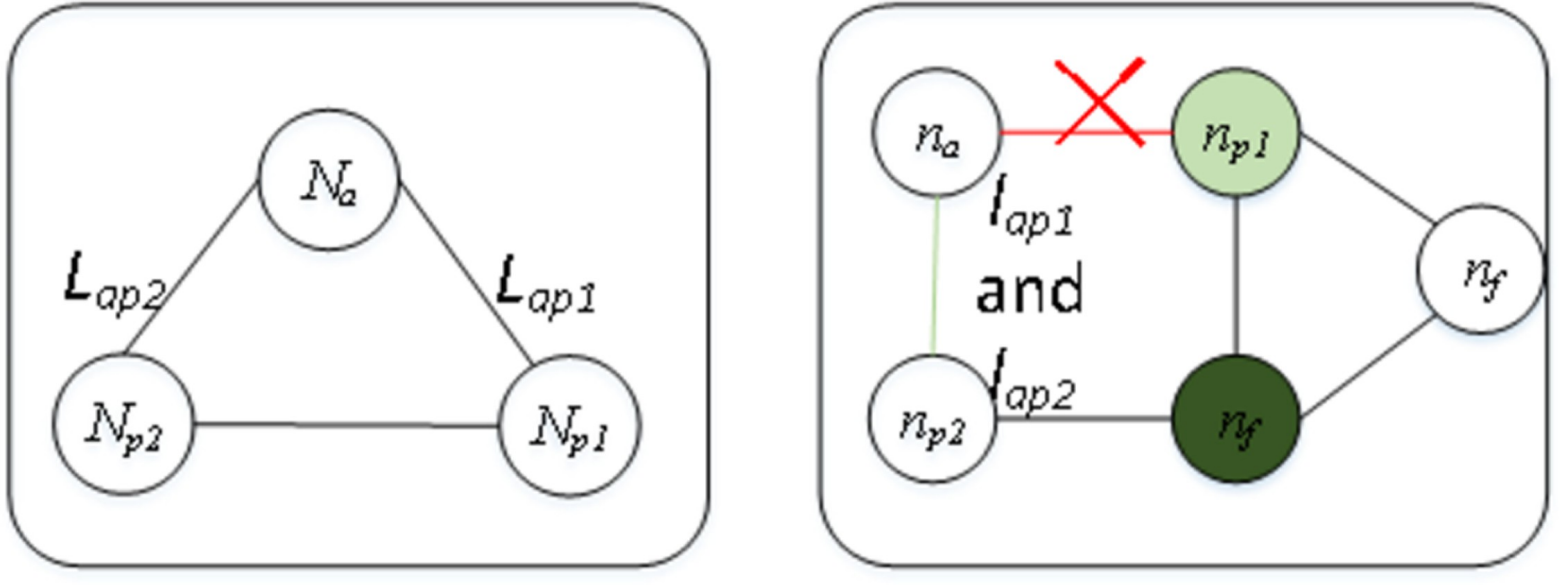
An example of mapping the virtual link *L*_*ap1*_ and *L*_*ap2*_ in *G*_*v*_ (left) on the same physical link *l*_*ap1*_ in *R*(*G*_*v*_) (right) because another adjacent link of node *N*_*a*_ (the red edge) does not satisfy the bandwidth constraint, while link *l*_*ap1*_ (the green edge) still has sufficient remaining bandwidth to host *L*_*ap1*_ even after hosting *L*_*ap2*_, and the adjacent node of *n*_*p2*_ (the dark green edge) has sufficient capacity to meet the demand of virtual node *N*_*p1*_.

**Fig 6:**
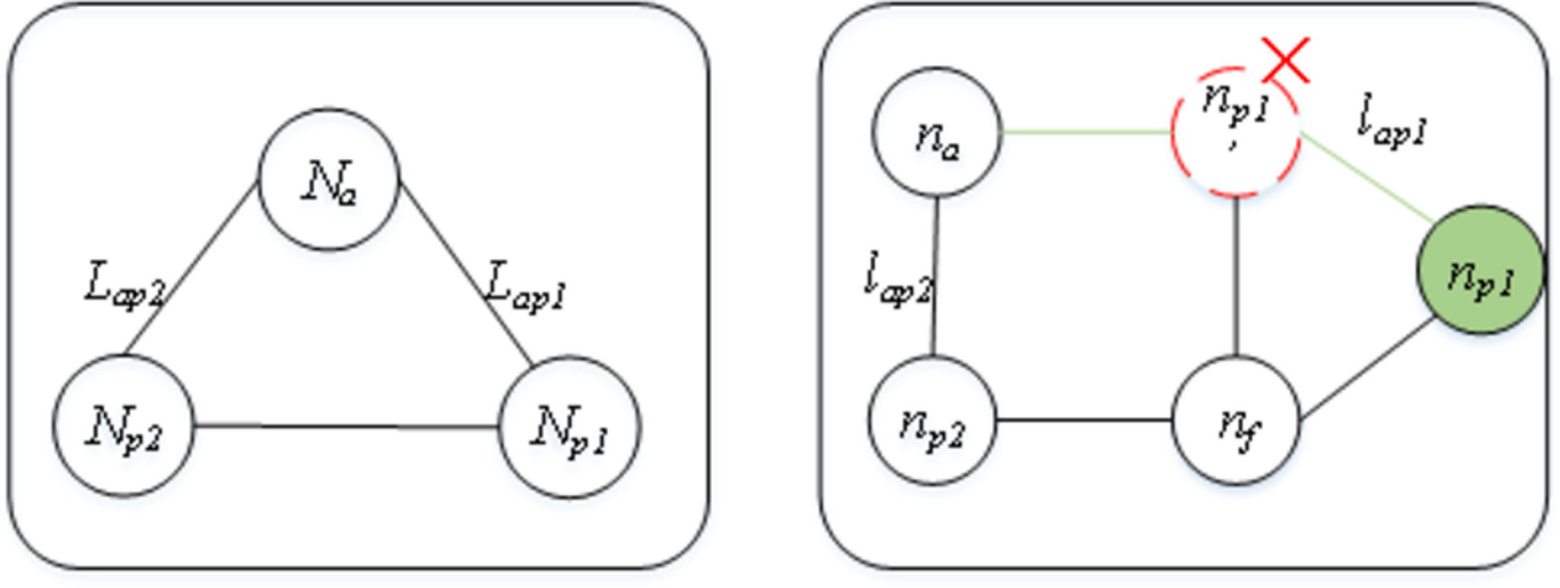
An example of mapping the virtual link *L*_*ap1*_ in *G*_*v*_ (left) on the physical link *l*_*ap1*_ in *R*(*G*_*v*_) (right) by extending the link to the next hop node *n*_*p1*_ (the green node) of node *n*_*p1*_’ because the remaining capacity of *n*_*p1*_’ does not meet the demand of virtual node *N*_*p1*_.

To facilitate understanding, an example of matching one virtual link in *G*_*v*_ to its counterpart in *R*(*G*_*v*_) is illustrated in Figs 4–6. The request graph is composed of three links and tree nodes, and the substrate graph is composed of six links and five nodes, in which a virtual node *N*_*a*_ is mapped to the physical node *n*_*a*_. Next, when matching the adjacency links *L*_*ap1*_ and *L*_*ap2*_ of *N*_*a*_ (Fig 4), if *l*_*ap1*_ satisfies all constraints of *L*_*ap1*_, i.e., both the bandwidth and delay of *l*_*ap1*_ meet the *L*_*ap1*_ requirements, and *n*_*p1*_ at the other end of *l*_*ap1*_ also meets the capacity requirements of virtual node *N*_*p1*_, then we match *L*_*ap1*_ to *l*_*ap1*_. Similarly, if *L*_*ap2*_ meets all the requirements of *l*_*ap2*_, we match *L*_*ap2*_ to *l*_*ap2*_. However, if the bandwidth constraint cannot be met, then we match *L*_*ap1*_ to another adjacent link of *N*_*a*_ with sufficient bandwidth (Fig 5), *l*_*ap2*_ is split to host *L*_*ap1*_ and *L*_*ap2*_, and *N*_*p1*_ is matched to an *n*_*p2*_ adjacent node that satisfies the *N*_*p1*_ capacity constraint. If the link’s resource constraint is satisfied, but the capacity of the node at the other end of this link is not satisfied (Fig 6), then we match *N*_*p1*_ to an adjacent node of *n*_*p1*_’ that meets the capacity constraint.

Next, Eq (15) is used to calculate the editing distance *g*_*vne*_(*f*) during the editing process of the substructure isomorphism of *G*_*v*_ into that of *R*(*G*_*v*_), that is, the energy consumption of the physical resources turned on in *R*(*G*_*v*_) to hold this virtual request.

Finally, we edit the remaining part of *G*_*v*_ excluding the common substructure isomorphism to *R*(*G*_*v*_) by finding their shortest paths in the common substructure isomorphism and calculate the energy consumption of the physical resources that turn on in this region, denoted as *h*_*vne*_(*f*). Given that the remainder is only partial connection information between the nodes, to ensure the VNE service quality, we relax the restriction of embedding when editing this part: only the resource constraints are retained.

In general, using *R*(*G*_*v*_) to host VNRs provides an absolute advantage in controlling the resources usage by limiting *R*(*G*_*v*_) to a scale that is almost as small as *G*_*v*_ so that only a few of the necessary resources are opened. Moreover, it can also provide flexible and predictable services for users. Since the bounds of *R*(*G*_*v*_) are set by the resource constraints and the structure constraints, we can make a new bound for *R*(*G*_*v*_) by changing the constraints in the restrictions and find a new *R*(*G*_*v*_) to host *G*_*v*_ while fitting the new requirement.

### Searching the highest potential resource management region

Different from the previous metaheuristic algorithm that traverses the solution space with a fixed population [28,29]. SMO is an algorithm that simulates the intelligent foraging behavior of spider monkeys with a fission-fusion social structure (FFSS). In FFSS, a swarm is a social grouping pattern in which individuals form temporary subgroups whose members belong to a larger unit group. There can be fluid movement between subgroups and unit groups such that group composition and size change frequently and vice versa based on the real-time situation of the search to control the optimization speed. Therefore, when we search for the highest potential *R*(*G*_*v*_) in a parallel way in the SN, we take advantage of this feature of SMO to converge to a potential physical region quickly by searching with a small-size group in the SN at the beginning. When the research is stagnant, we expand the population size by splitting the group into a larger group to explore and repeat the previous search phases until we find the near-optimal solution. Therefore, during this optimization process, we can balance the exploration and exploitation capability of the optimization algorithm while maintaining the convergence speed.

**Fig 7:**
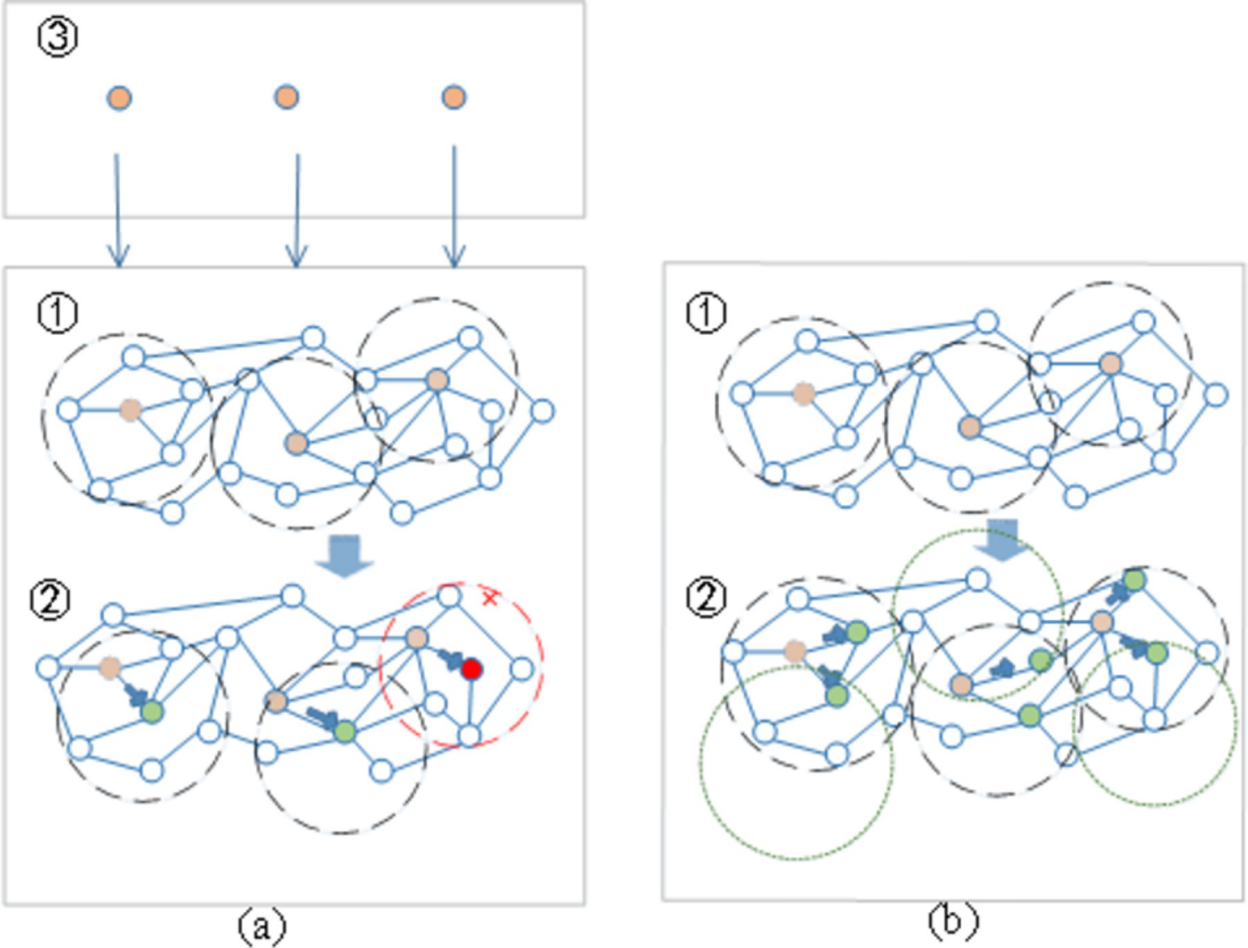
Updating the *SM* positions: (a) *SM* position updates in the subgroup. The *SM* marked with a red cross represents the region where VNR requirements are not met and must be pruned; (b) the population is divided into smaller groups with the *n*_*source*_ placed in two positions.

**Fig 8:**
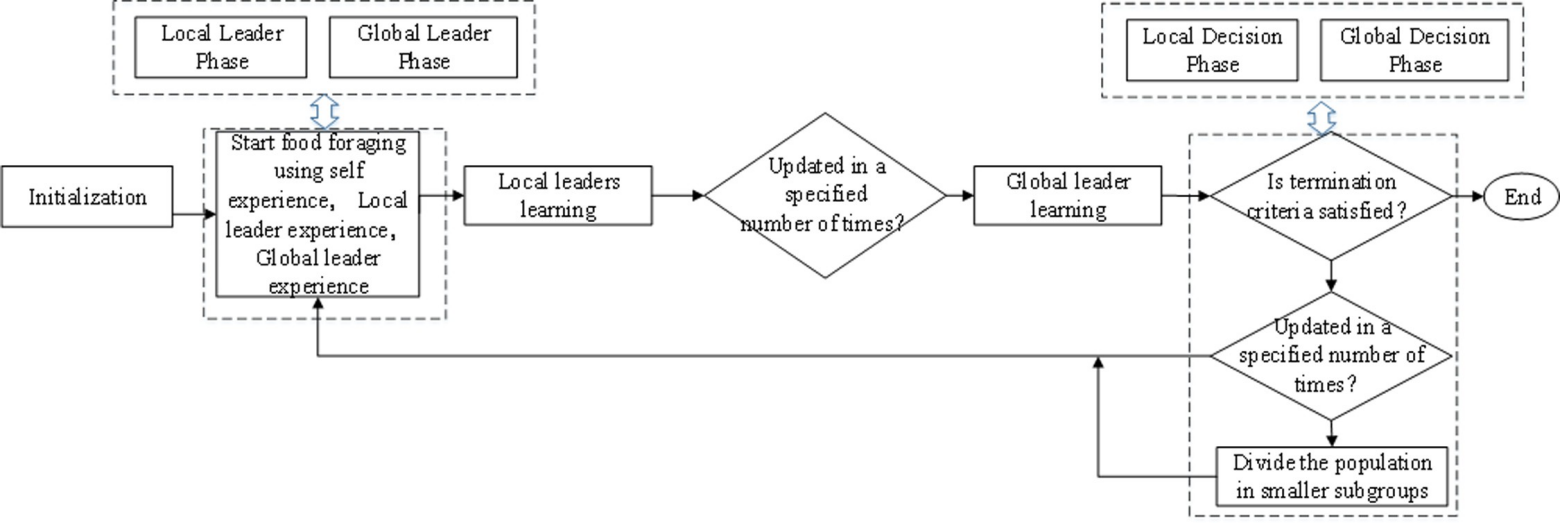
SMO-based optimization process.

There are six stages in the algorithm: the local leader phase, global leader phase, local leader learning phase, global leader learning phase, local leader decision phase and global leader decision phase. The first and second phases are used to explore the search region while generating the new positions, i.e., the new solutions to this problem, for all the group members by using self experience, local/global leader (the spider monkey with the greatest fitness in its subgroup/unit group) experience and group member experience to promote the exploitation. The third and fourth phases, are used to determine whether the local best and global best solutions are updating in a predefined number of trials. If not, then the solution is considered stagnated. The fifth and sixth phases are used to avoid the stagnation or premature convergence of the local solutions/global best solution and control the population size. If the local best solution is not updated in a predefined number of trials (*LocalLeaderLimit*) then all the members of that group are reinitialized. If the global best solution is not updated within a predefined number of trials (*GlobalLeaderLimit*) then the group is divided into smaller subgroups. The position update process of spider monkeys (*SM*s) is shown in Fig 7. Moreover, to clarify how we use and enhance SMO to find the highest potential *R*(*G*_*v*_), a step-by-step scenario graph describing the overall process and the pseudocode of VNE_MR are shown in Fig 8 and Algorithm 2 respectively. The implementation process is explained below:

**Algorithm 2** VNE_MR

Input: *G*_*s*_ and *G*_*v*_, *P*, *MG*, *local leader limit*, *global leader limit*, *pr*.

**1:** calculate the *N*_*source*_ *of G*_*V*_ use (23);

**2:** randomly select *P n*_*sourcei*_ (*i = 1* …*P*);

**3:** for each *n*_*sourcei*_

**4:**  call Algorithm 1 to delimit *R*_*i*_(*G*_*v*_) corresponding to the *SM*_*i*_

**5:**  use (22) to calculate *SM*_*i*_’s fitness

**6:** Select global leader and local leaders by applying greedy selection

**7: while** (termination criteria is not satisfied) **do:**

**8:  for** *k* in *MG* **do:**

**9:**    generate a new position for each *SM*_*i*_∈*k*th subgroup using (18)

**10:**    produce new position for each *SM*_*i*_∈*k*th subgroup using (19)

**11:**    update global leader and local leaders; record the position and the fitness

**12**: call Algorithm 3 to update all the groups

**13:** **end while**

#### Initialization

Initially, we generate a population of *P* spider *SM*s as the VNE solutions, i.e., delimit *P R*(*G*_*v*_) in the substrate graph. *SM*_*i*_ represents the *i*th *SM* in the population, i.e., *i*th *R*(*G*_*v*_). The local/global leader represents *R*(*G*_*v*_) with the greatest fitness in its subgroup/swarm and *MG* represents the maximum number of groups in the swarm. However, as this optimization process starts with a single group having all the *SM*s, in the beginning, the local leader and global leader are both the same *SM*. Moreover, because the goal of this study is to find the most energy-saving *R*(*G*_*v*_), each *SM*_*i*_ corresponds to the *i*th *R*_*i*_(*G*_*v*_). Therefore, the fitness of each *SM*_*i*_ is equal to the energy consumption of physical resources opened in *R*_*i*_(*G*_*v*_):

$$Fitness\;=E\left(R\left(G_{V}\right)\right)=\sum_{n\in R(GV)}E(n)+\sum_{l\in R(GV)}E(l)$$ (22)

In addition, according to the previous section, the following conclusion can be easily drawn: to build an energy-saving *R*(*G*_*v*_) with a compact structure in the SN, a proper *source node* is essential. Therefore, to ensure *R*(*G*_*v*_) has a compact structure, the region must have short links, and a high utilization rate is high; thus, Eq (23) is presented to calculate the virtual node *N*_*source*_ of *G*_*v*_, which is the node with the shortest distance sum from all other nodes in the graph; the formula for calculating *N*_*source*_ is:

$$N_{source}\;=\text{Min}\left(\text{sum}\;\left(\text{hops}\;\text{from}\;\text{other}\;\text{nodes}\;\;\text{to}\;\text{the}\;N_{source\;}\right)\right)$$ (23)

Then, *P* physical nodes, which are the candidate mapping nodes of *N*_*source*,_ are randomly selected in the SN and each physical node is marked as *n*_*sourcei*_ (*i = 1* … *P*) to build the *R*(*G*_*v*_) (Fig 7(A): 3→1). A detailed description is provided in Lines 1–5 of Algorithm 2.

#### Local leader phase (LLP) and global leader phase (GLP)

To accelerate the convergence speed, in the LLP, every *SM* generates its current position based on the experience information of the local leader as well as local group members. The fitness value of the obtained new position is calculated. The position update equation for the *i*th SM (which is a member of the *k*th subgroup) is:

$$SM_{newi\;}=SM_{i}⊕\left[r_{1}\cdot \left(LL_{k}\Theta SM_{i}\right)+r_{2}\cdot \left(SM_{r}\Theta SM_{i}\right)\right]$$ (24)

where *LL*_*k*_ represents the *k*th subgroup leader position.

*SMr* is the *r*th *SM*, which is chosen randomly within the *k*th group such that *r ≠ i*. *r*_1_ and *r*_2_ represent the two random variables that are uniformly distributed in [0, 1] and *r*_1_ + *r*_2_ = 1. As VNE is a discrete problem, numeric computing is divided into taking one step (in one-hop units) toward *LL*_*k*_ or *SM*_*r*_. As shown in Fig 7(A), after we calculate the updating direction of each *SM*_*i*_ (*i* = 1,2,3) by using the contents in the brackets of (17), we move *SM*_*i*_ from the original position (black dotted circle in Fig 7(A) ‐ ➀) to this direction by moving the center node *n*_*source*_ of *R*_*i*_(*G*_*v*_) (yellow node in Fig 7(A) ‐ ➁) to the calculated direction by one hop (green node in Fig 7(A) ‐ ➁)). Θ denotes the AND operation. ⊕ denotes the calculation of the shortest path between two *n*_*source*_ of *R*(*G*_*v*_)).

In the global leader phase, all *SM*s update their position using the experiences of the global leader and local group members. The position update equation for this phase is as follows:

$$SM_{newi\;}=SM_{i}⊕\left[r_{1}\cdot \left(GL\;\Theta \;SM_{i}\right)+r_{2}\cdot \left(SM_{r}\Theta SM_{i}\right)\right]$$ (25)

#### Global leader learning (GLL) phase and local leader learning (LLL) phase

The local leaders and global leader are updated in this phase by applying greedy selection in its subgroup and the population, respectively. Furthermore, in the LLL phase, we check whether the position of the local leader in its group is updating, and if not, then the *local limit count* is incremented by 1. In the GLL phase, we check whether the position of the global leader in the whole population is updating, and if not, then the *global limit count* is incremented by 1.

#### Local leader decision (LLD) phase and Global Leader Decision (GLD) phase

If any local leader position is not updated to the threshold *local leader limit*, then the members in that subgroup update their positions either by random initialization or by collective information from the global leader and local leader through (19), based on the *pr*. The pseudocode of this phase for the *k*th group is shown in Lines 1–8 of Algorithm 3.

$$SM_{newi\;}=SM_{i}⊕\left[r_{2}\cdot \left(GL\;\Theta \;SM_{i}\right)+r_{3}\cdot \left(SM_{i}\;\Theta \;LL_{k}\right)\right]$$ (26)

where *LLC*k is the trial counter for the local best solution of the *k*th group.

In the global leader decision phase, if the position is not updated to the threshold *global leader limit*, then the population is divided into smaller groups (Fig 7(B)). At first, the population is divided into two groups, and then three groups, etc.; each time, the local leaders in the newly formed groups are elected until *MG*’s limit is reached. In the case in which the maximum number of groups is formed, then the global leader combines all the groups to form a single unit group and further selects the position with the highest fitness as the solution to the problem. The operation in this phase is shown in lines 9–15 of Algorithm 3.

**Algorithm 3** Local leader decision and global leader decision phase

**1:  for** *k*th group in *MG* **do:**

**2:     if** *LLC*_*k*_ > *local leader limit* **then**

**3:**      *LLC*_*k*_ = 0

**4:      for** *SMi* in the *k*th group do

**5:**        if *U*(0,1)≥ *pr* **then**

**6:**          random select a *n*_*source*_ from the substrate network and initialize *SM*_*i*_

**7:**        else

**8:**          initialize *SMi* using (19)

**9:  if** *global limit count* > *global leader* **lthen**

**10:**    *global limit count* = 0

**11:    if** number of groups < *MG* **then**

**12:**      divide the population into subgroups.

**13:**    **else**

**14:**      combine all the groups to make a single group.

**15:**   Update Local Leader’s position.

Finally, for SMOs to play a better role in VNE problems, several points must be noted.

First, traversing the substrate network to find the optimal region quickly is a discrete problem that needs to be solved. To speed up convergence, we set every *SM* to use its self-experience, leader experience and group members experience to update its position in the LLP and GLP instead of having a certain probability to update.

Second, during the optimization process, if the capacity of a selected *n*_*source*_ does not meet the requirements of its corresponding virtual node *N*_*source*_, this subgroup to which it belongs is pruned (Fig 7(A)). Regardless of which direction we select on the update, we must obey resource and structure restrictions as the proposed bounds to achieve rapid pruning from the surrounding physical topology. After all, if one constraint is not satisfied, then this *R*(*G*_*v*_) cannot be used, and the next endeavor will be meaningless.

Third, the search speed will be faster with a large population; however, gaining this advantage may sacrifice some computational time. If there are *N SM*s in the population, then the time complexity of VNE_MR is *o*(|*N*_*v*_| + |*L*_*v*_| + |*L*_*R*(*Gv*)_| + |*N*_*R*(*Gv*)_|^2^) ⋅|*P*|, where |*N*|. represents the number of nodes, |*L*| represents the number of links, and |*P*| represents the total number of *SM*s used in this algorithm, which has a direct effect on the computational time. Therefore, initially, there is a small group in our algorithm so every newly generated position is attracted toward the best position; the population is only divided into smaller subgroups to expand exploitation when regeneration stagnates.

## Performance evaluation

In this study, we designed two sets of experiments to demonstrate the resource management ability of the proposed *VNE_MR* algorithm. In the first group, to verify the overall performance of VNE_MR, VNE_MR is compared to VNE_TAGRD [14] and OPaCoVNE [16], all of which have the same goal: to solve the virtual network embedding problem using fewer resources and consuming less power. Table 1 provides a high-level comparison of VME_MR and those methods, listing their strategies and resource constraints.

**Table 1:** Comparing VNE_MR to VNE_TAGRD and PaCoVNE.

Item	VNE_MR	VNE_TARGET	OPaCoVNE
Embedding Strategy	building a resource management unit by delimiting an *R*(*G*_*v*_) similar in size to *G*_*v*_	focusing on the availability of resources	building a resource management unit by treating pairs of nodes and their edge as a segment
Power consumption	Yes	Yes	Yes
End-to-end delay	Yes	Yes	Yes

In the second group, VNE_MR is compared to two metaheuristic-based VNE algorithms, namely EE_CTA [26] and MO-NPSO [25], to verify that our optimization method design based on SMO can successfully keep the optimization cost within a small value while maintaining adequate exploration of the substrate network to ensure the quality of service. In addition, VNE_MRWD is a VNE algorithm without delay requirements, which is designed as VNE_MR’s comparison experiment. It is presented to delimit a new *R*(*G*_*v*_) by changing the resource constraint model introduced in Section 4.1 and then solving the VNE problem by using the improved SMO to find the highest potential *R*(*G*_*v*_) to host *G*_*v*_. Therefore, it is designed to verify the flexibility of VNE_MR. Table 2 provides a high-level comparison between VNE_MR and those methods, listing their resource constraints and other parameters.

**Table 2:** Comparing VNE_MR to VNE_MRWD and EE_CTA.

Item	VNE_MR	VNE_MRWD	EE_CTA	MO-NPSO
Optimization strategy	Based on the SMO	Based on the SMO	Based on the Non-dominated Sorting Genetic algorithm	Based on the particle swarm optimization algorithm
Power consumption	Yes	Yes	Yes	Yes
End-to-end delay	Yes	No	No	No

### Simulation settings

Substrate network and virtual network topologies were randomly generated using the GT-ITM tool, and the mean probability of a pair of two nodes being connected was set equal to 0.5. The substrate network included 100 nodes, *CPU* and *BW* resources were given as real numbers uniformly distributed between 50 and 100, and delay in each substrate edge was randomly selected between 1 and 25. The number of virtual nodes per VNR was randomly determined by a uniform distribution between 2 and 10. The virtual *CPU* and *BW* resources were real numbers uniformly distributed between 0–20 and 0–50, respectively, while delay in each virtual edge was randomly selected between 20 and 100. The VNR arrival process was simulated as a Poisson process, and its average arrival rate was set to 5 VNRs per 100 time units. Its residence time obeyed an exponential distribution with an average value of 500 time units. To map a sequence of 2,000 VNRs, our simulation lasted for approximately 40,000 time units. A time window was equal to 2,000 time units. To obtain final representative and stable experimental results, we ran all of our simulations in 20 time windows and collected the experimental data after 5 time windows. To ensure acceptable convergence of the SMO used in VNE_MR, we investigated the values of best and average fitness in different generations. The parameters used in the SMO were *P* = 5, *MG* = 3, *local leader limit* = 2, *global leader limit* = 3, and *pr* = 0.9.

### VNE performance metrics

The comprehensive ability to manage the resources of the VNE problem can be judged in terms of the following metrics [30].

1. Average number of open nodes (*N*_*ON*_):

$$N_{ON}=\frac{\sum_{i=1}^{nT}no_{i}}{n_{T}}$$

where *n*_*T*_ represents the number of all valid time periods from 0 to T and *n*_*Oi*_ represents the number of physical nodes that are active in effective period *i*.

2. The average number of open links (*N*_*OL*_) is

$$N_{OL}=\frac{\sum_{i=1}^{nT}lo_{i}}{n_{T}}$$

where *l*_*Oi*_ represents the number of open substrate links at time period *i*.

3. The average utilization of CPUs (*U*_*CPU*_) is

$$U_{CPU}=\frac{\sum_{i=1}^{nT}n_{\text{CPUi}}}{n_{T}}$$

where *n*_CPUi_ represents the CPU utilization of the node resources at time slice *i*.

4. The average utilization of bandwidth (*U*_*BW*_) is

$$U_{BW}=\frac{\sum_{i=1}^{nT}l_{BW\text{i}}}{n_{T}}$$

where *l*_*BW*i_ represents the bandwidth utilization of the link resources at time slice *i*.

5. The average energy consumption (*E*) is

$$E=\frac{\sum_{i=1}^{nT}\left(\sum_{N\in N_{v},n\in n_{s}}E(n)+\sum_{L\in L_{v},l\in l_{mn}}E(l)\right)}{nT}$$

where ($\sum_{N\in N_{v},n\in n_{s}}E(n)+\sum_{L\in L_{V},l\in l_{mn}}E(l)$) is the substrate resource consumption at time period *i*.

6. The average ratio of revenue and cost (*R*_*RC*_) is

$$R_{RC}=\frac{\sum_{i=1}^{AT}Revenue{\;}_{Ri}\;}{\sum_{i=1}^{AT}Cost{\;}_{Ri}}$$

where *A*_*T*_ represents the number of virtual networks that are received within the time period 0 to T and *Revenue*_*Ri*_ and *Cost*_*Ri*_ represent the revenue and cost, respectively, of the mapped virtual network *Ri*.

$$Revenue_{Ri}=\sum_{N\in N_{v}}C_{accept\;}(N)+\sum_{L_{MN}\in L_{v}}B_{accept\;}(L)$$

$$Cost_{Ri}=\sum_{nv\in N_{v}}x_{M}^{m}\cdot C(n)+\sum_{e_{v\in }E_{v}}\;x_{L_{MN}}^{l_{mn}}\cdot B(l)$$

where *C*_*accep*t_(*N*) represents the number of requested CPU resources for virtual node *N* that has been accepted and *B*_*accept*_(*L*) represents the amount of requested bandwidth for virtual link *L* that has been accepted.

### Discussion of VNE_MR’s overall performance

**Fig 9:**
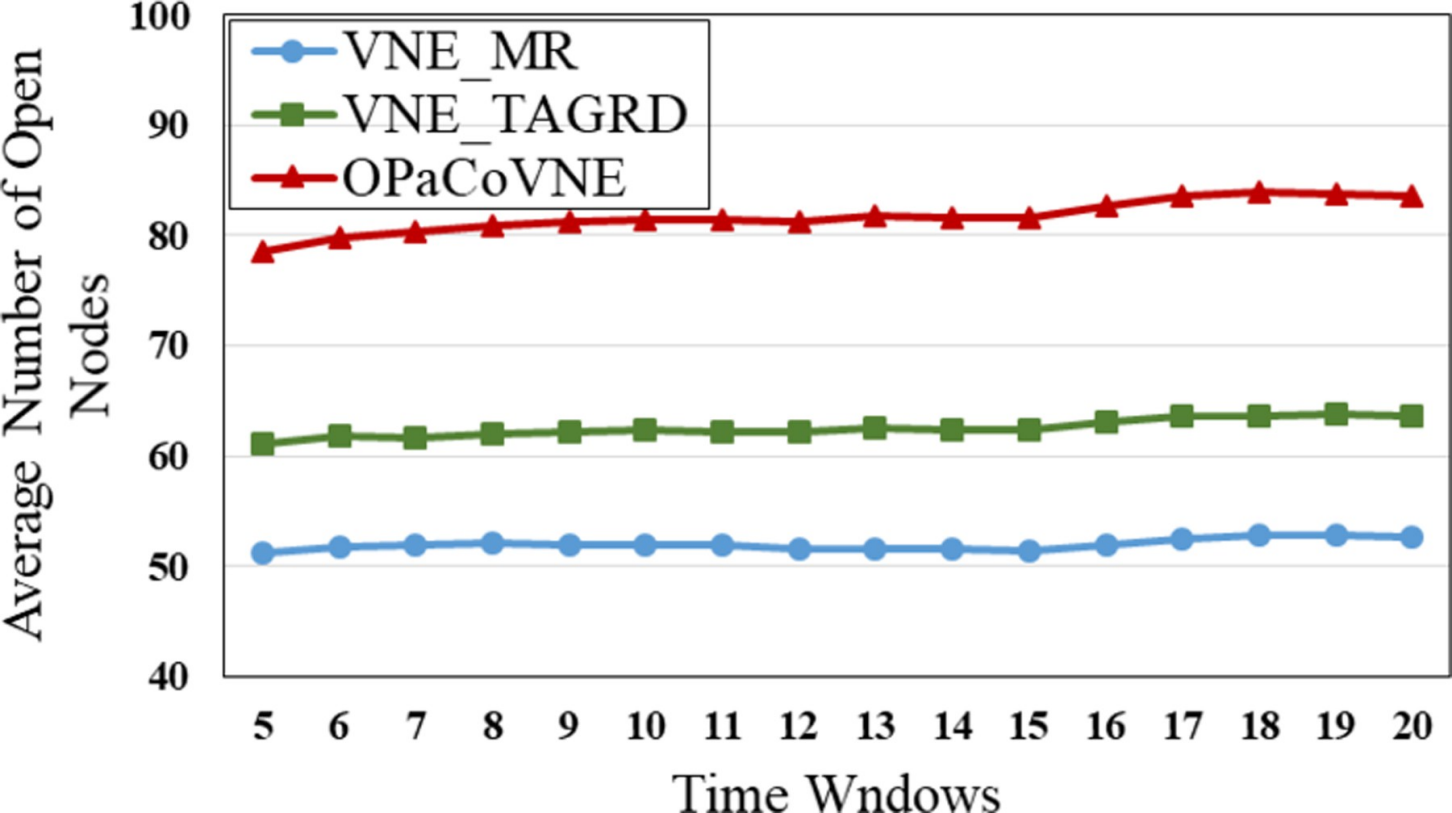
Average number of open nodes.

**Fig 10:**
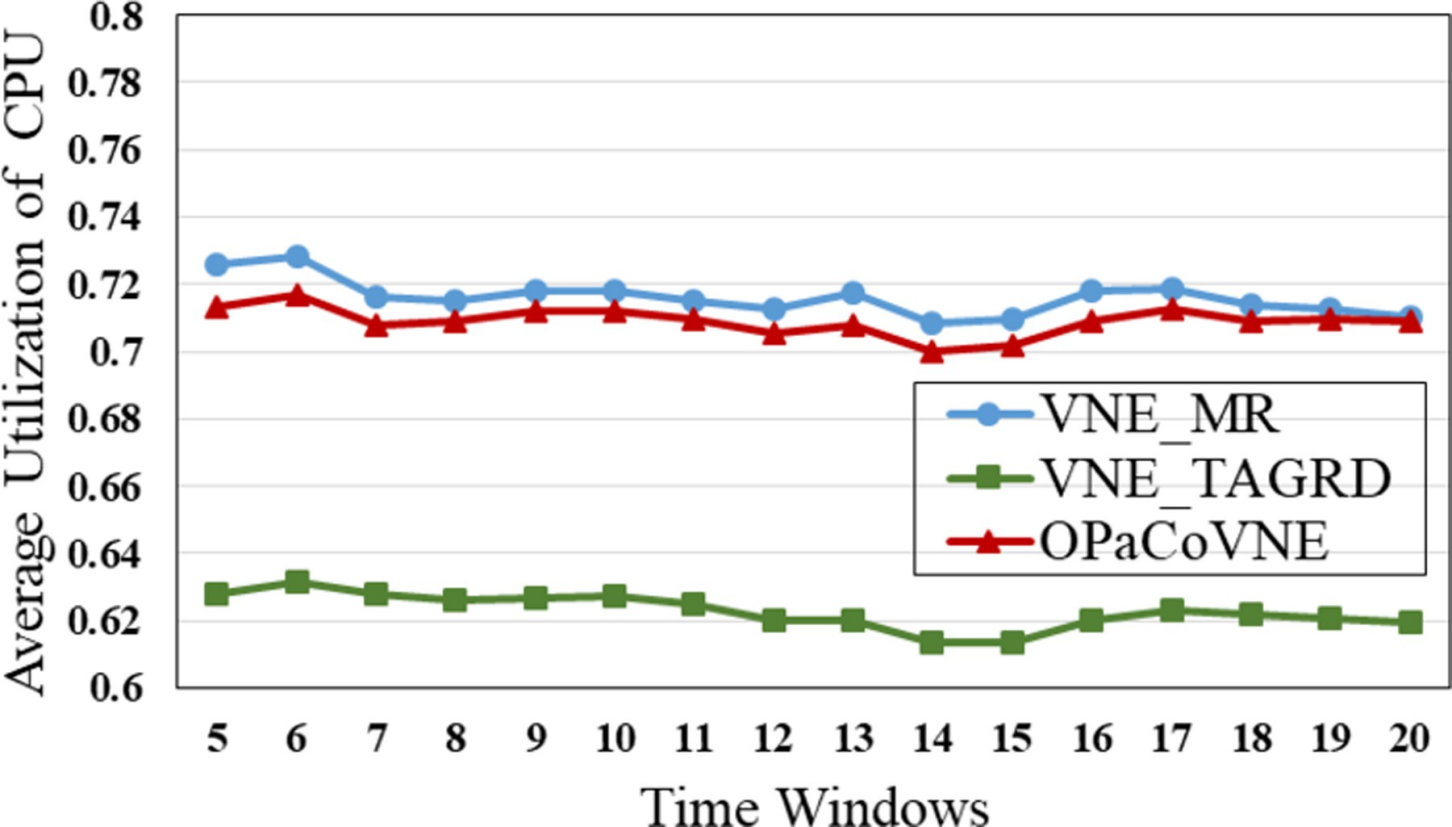
Average CPU utilization.

**Fig 11:**
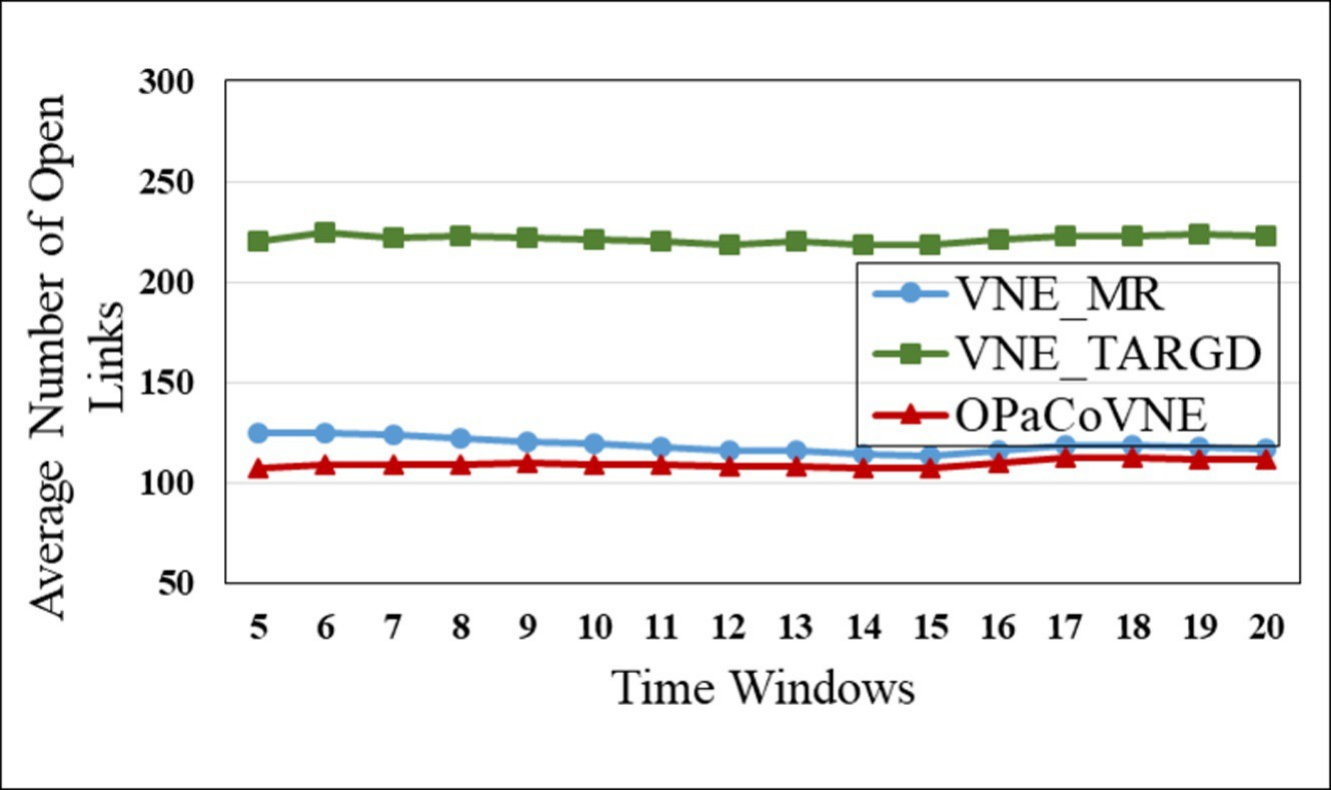
Average number of open links.

**Fig 12:**
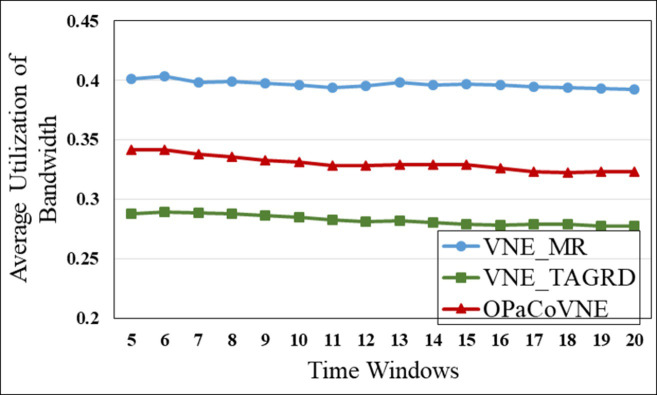
Average bandwidth utilization.

**Fig 13:**
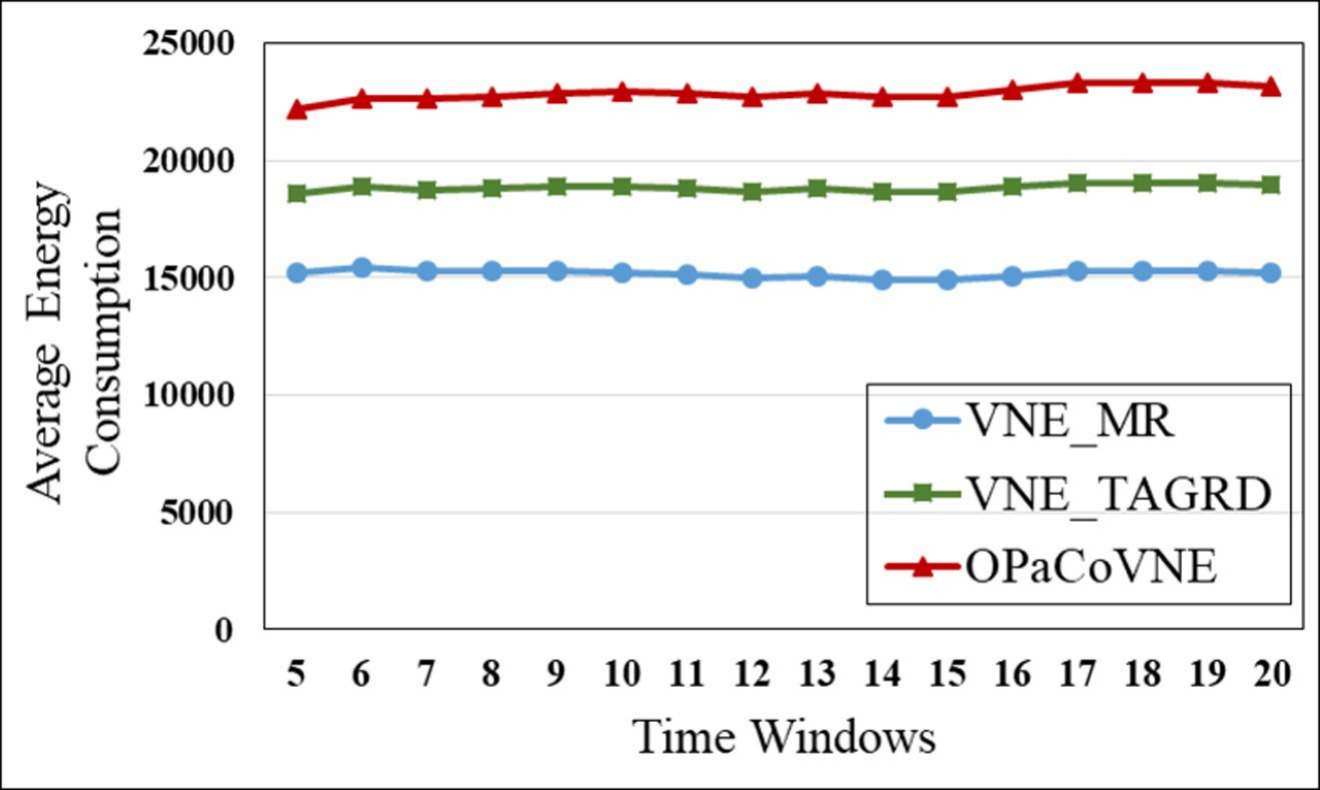
Average energy consumption.

In the first group, compared to other algorithms, VNE_MR has the smallest number of open nodes (≈50), indicating that the proposed algorithm achieves good results in node opening control (Fig 9). We calculate node utilization while collecting the number of open nodes (Fig 10). The VNE_MR node utilization rate (73%) exceeds those of the other algorithms (VNE_TAGRD ≈ 62%, OPaCoVNE ≈ 70%). As shown in Fig 11, after 5 time windows, the number of open links with VNE_MR is only 100 units, while that with VNE_TAGRD is 230 units, which is more than twice as high. The link resource utilization rate (40%) of VNE_MR is much higher than those of the other algorithms (VNE_TAGRD ≈ 28%, OPaCoVNE ≈ 32%) (Fig 12). According to Propositions 5 and 6, we can conclude that these performances directly affect the final average energy consumption (Fig 13).

**Fig 14:**
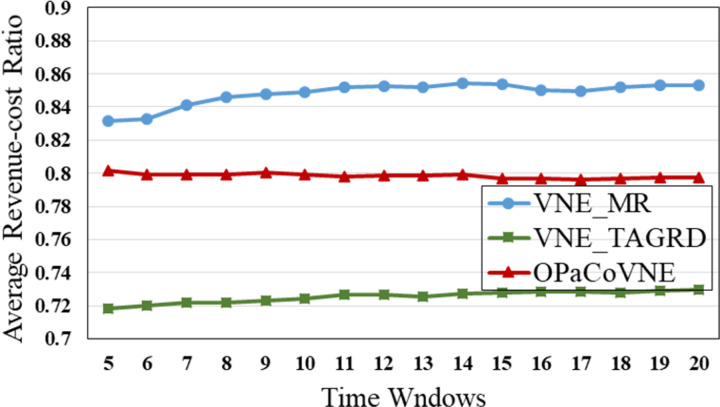
Average revenue-cost ratio.

This result likely occurs because VNE_TAGRD, a two-stage algorithm, only focuses on resource availability and ignores the coordination between nodes and links. Nodes selected in this manner may be scattered, which results in the use of unnecessary substrate resources. OPaCoVNE manages resources in several resource management units, named segments so that the original topology structure of*G*_*v*_ is disturbed when requirements are allocated in these segments. As the nodes and links are firmly bound in the segment, flexibility and fault tolerance are markedly reduced. Thus, these segments can only be separated to find the appropriate physical resource, which leads to opening hidden resources. Fig 13 shows the energy consumption results. The energy consumption of VNE_MR exhibits an apparent advantage over other algorithms. In addition to the energy savings shown, the ratio of revenue and cost is another advantage of VNE_MR (Fig 14). The ratio is maintained at 86%, and only 50 nodes and 100 links are turned on. The energy consumption is also the lowest among all of the tested algorithms because we use an edge-based graph edit distance method to edit *G*_*v*_ to the physical *R*(*G*_*v*_) in the substrate graph, keeping the size of *R*(*G*_*v*_) similar to that of *G*_*v*_, so that when mapping a VNR, only the minimum network resources need to be turned on, and no hidden resources are used. In addition, if this direct approach fails to find the optimal physical region in the SN, we also designed a metaheuristic optimization method based on SMO to find the highest potential *R*(*G*_*v*_) in a parallel manner.

**Fig 15:**
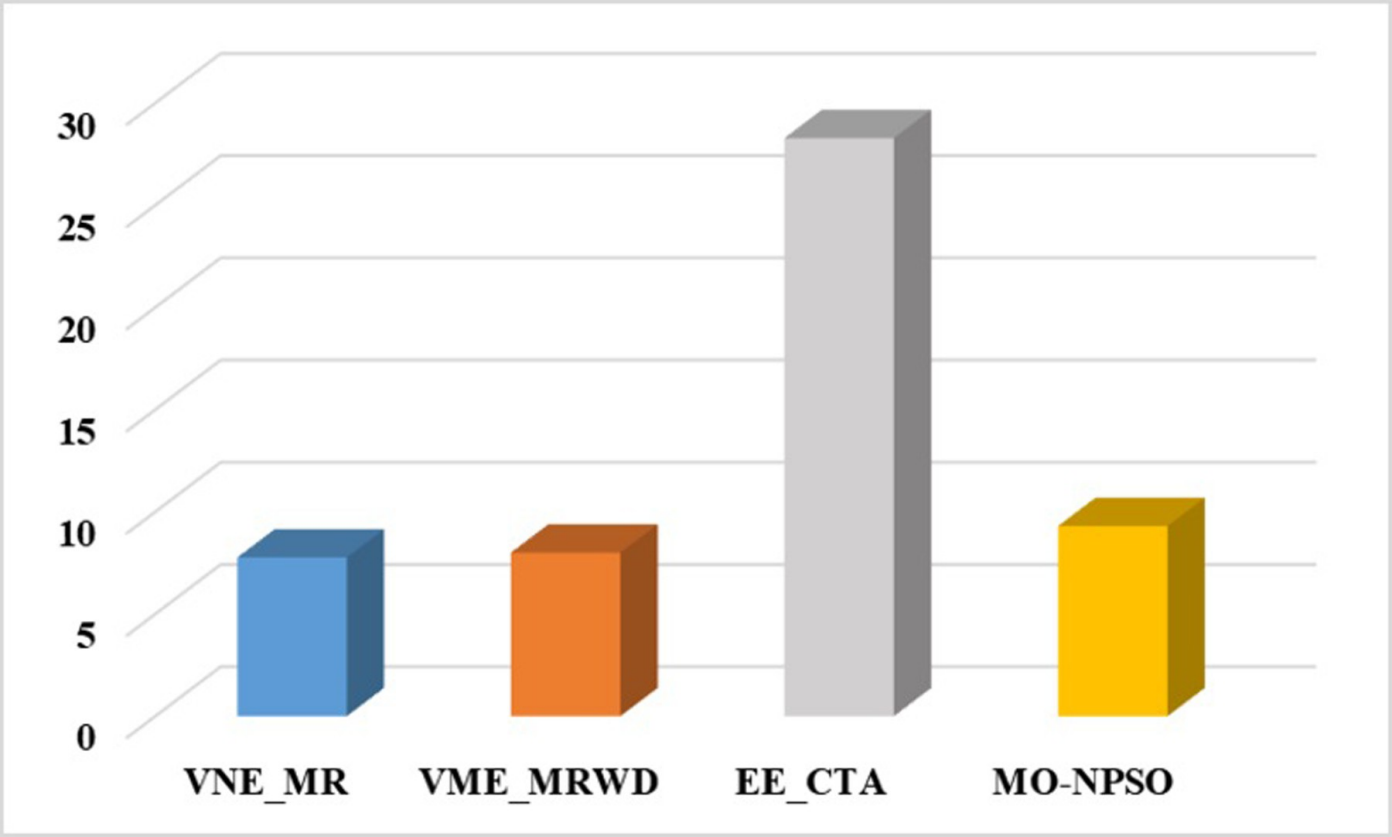
Average processing time.

**Fig 16:**
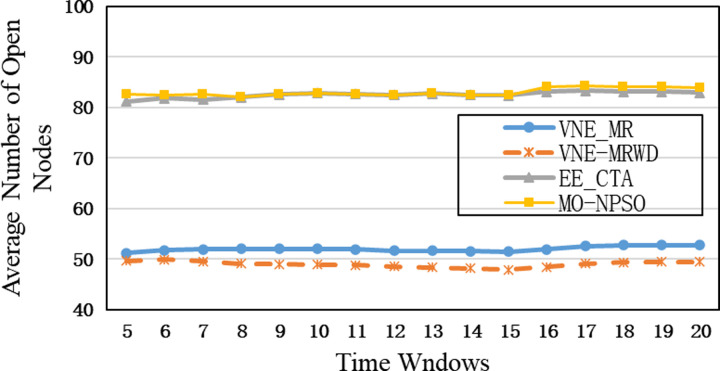
Average number of open nodes.

**Fig 17:**
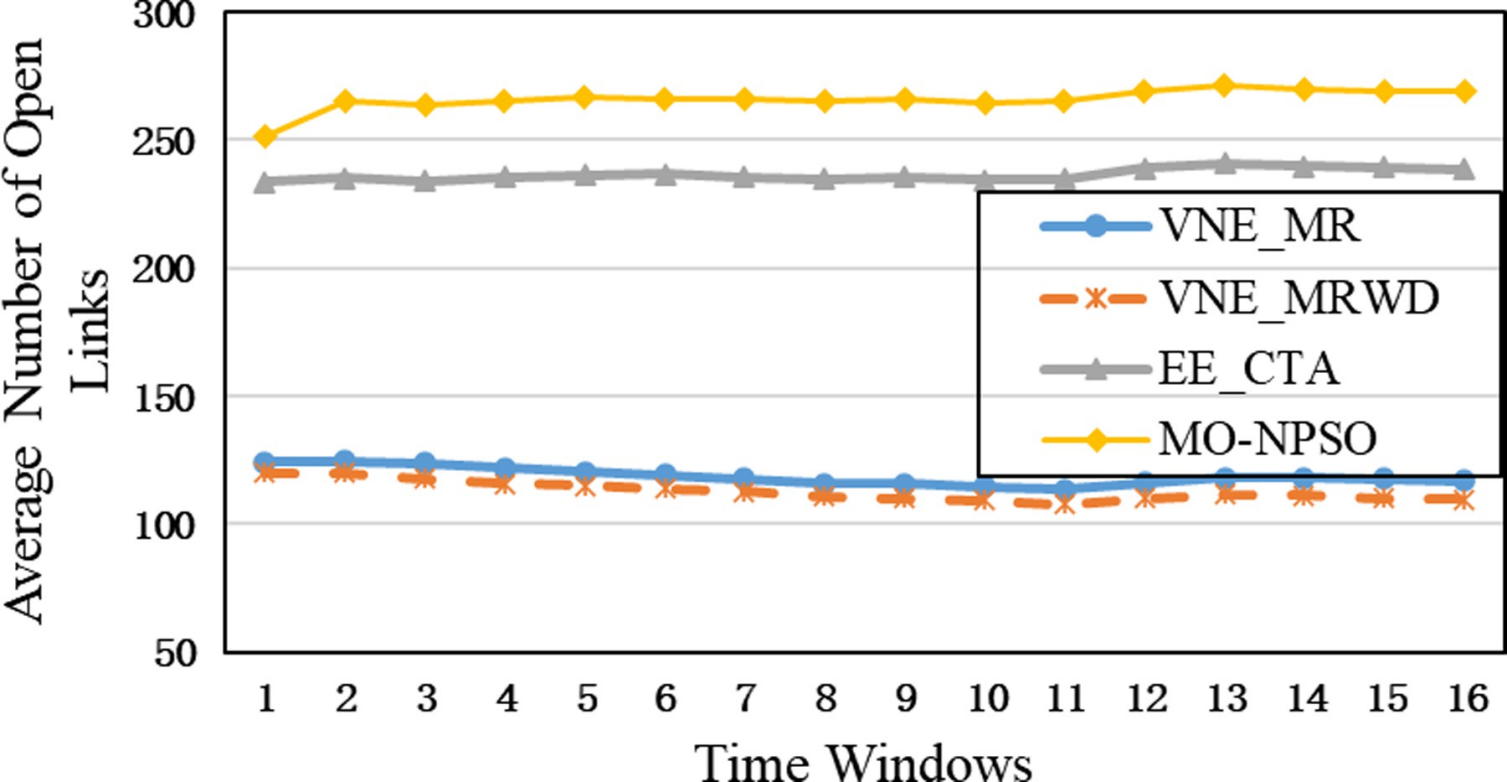
Average number of open links.

**Fig 18:**
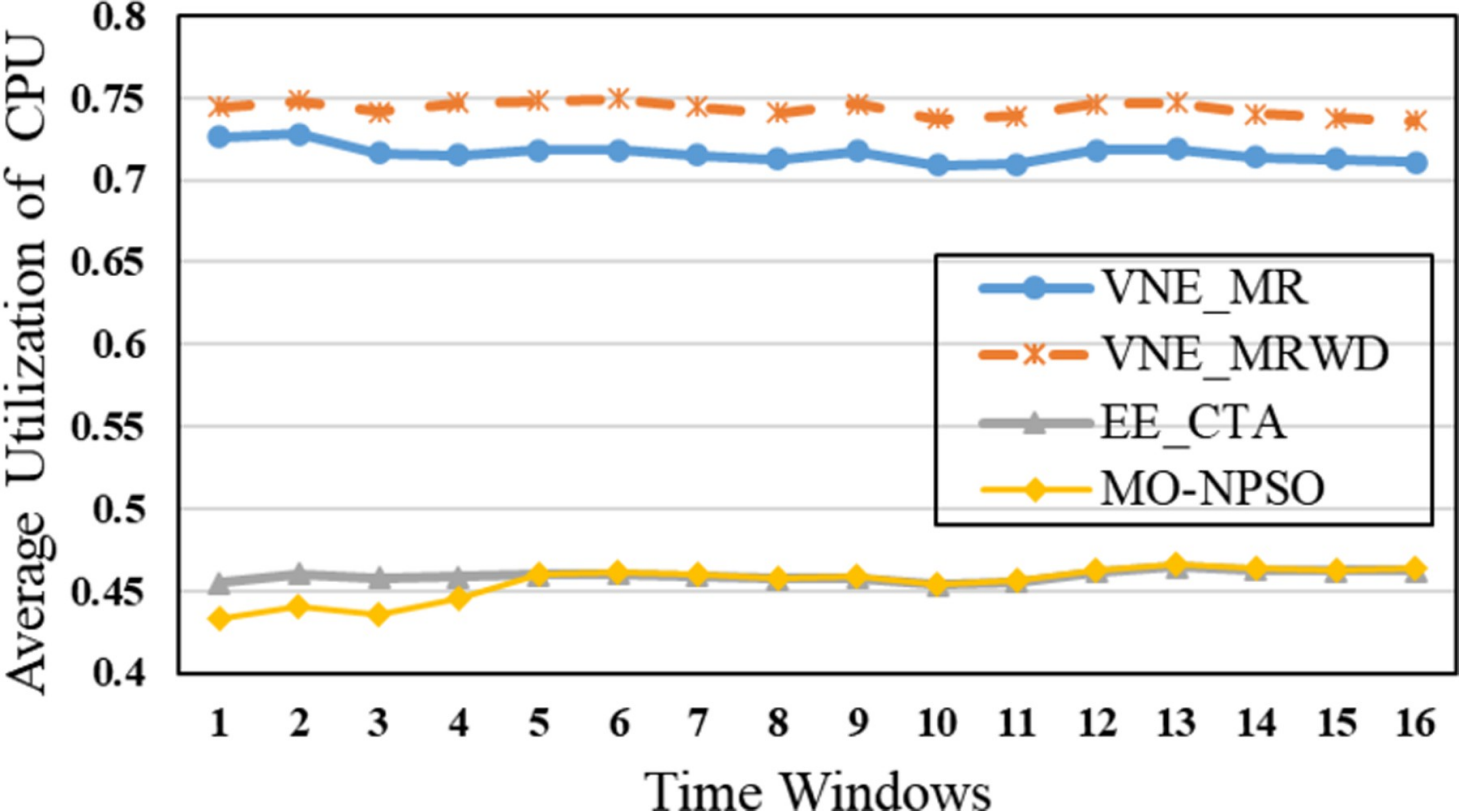
Average node utilization.

**Fig 19:**
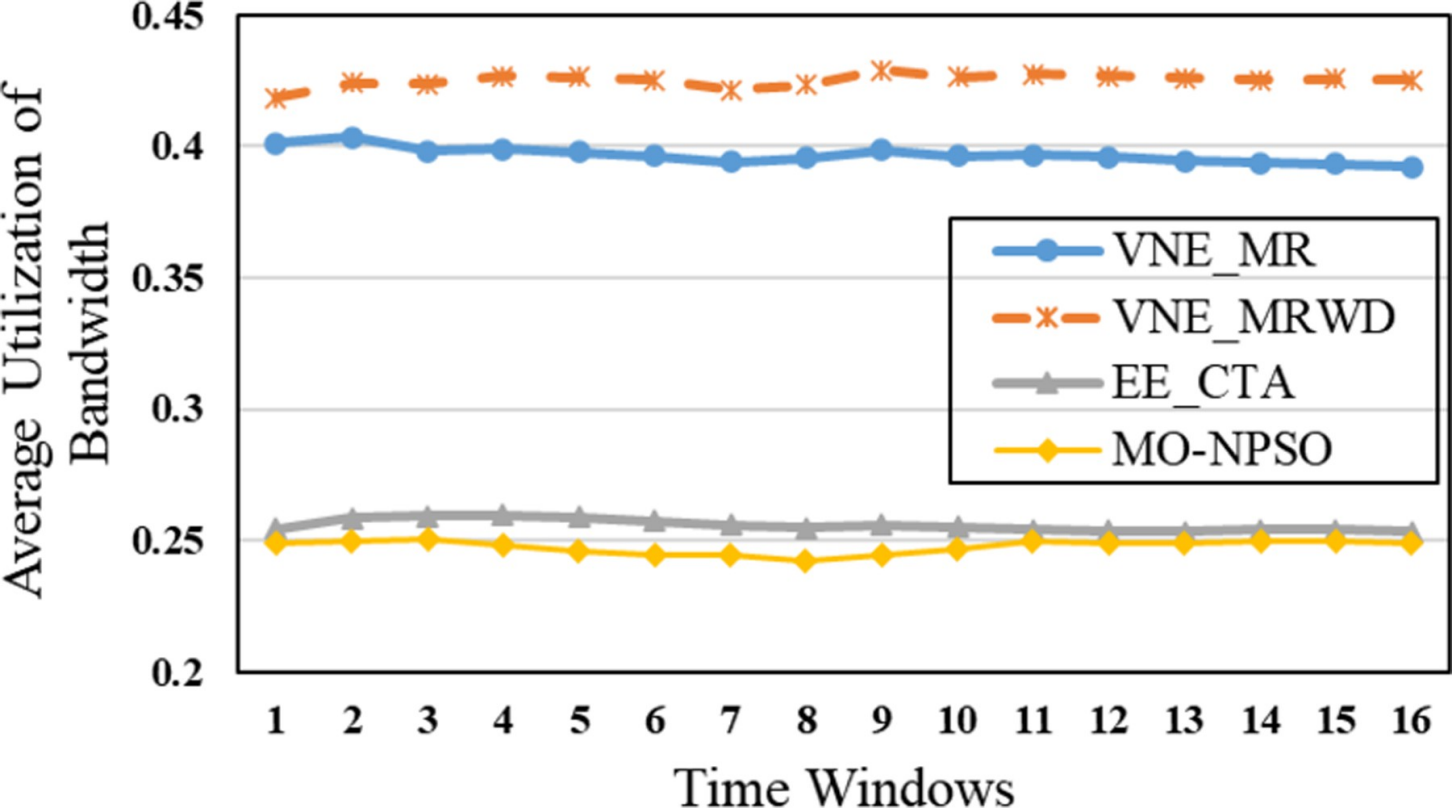
Average bandwidth utilization.

**Fig 20:**
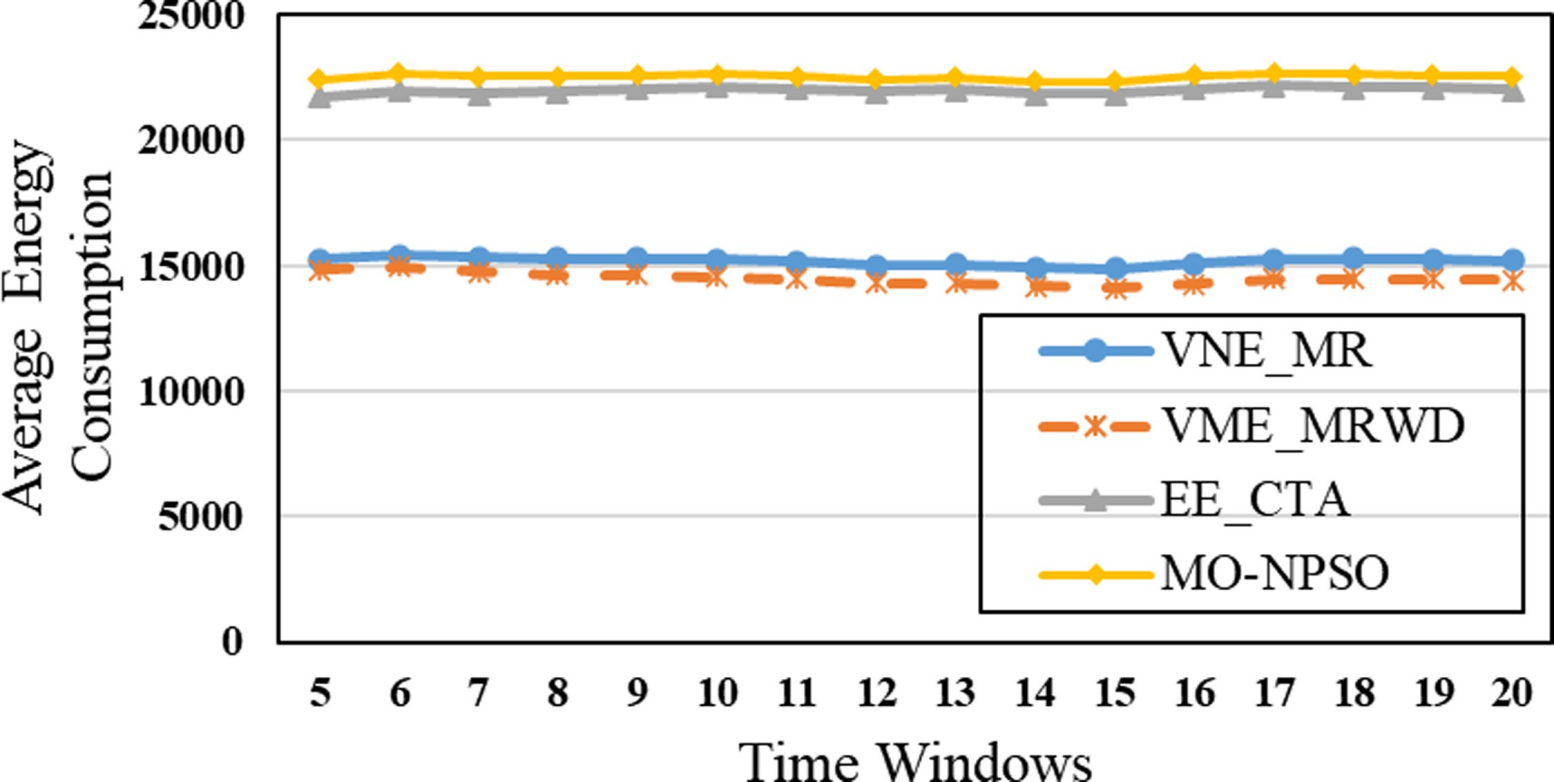
Average energy consumption.

**Fig 21:**
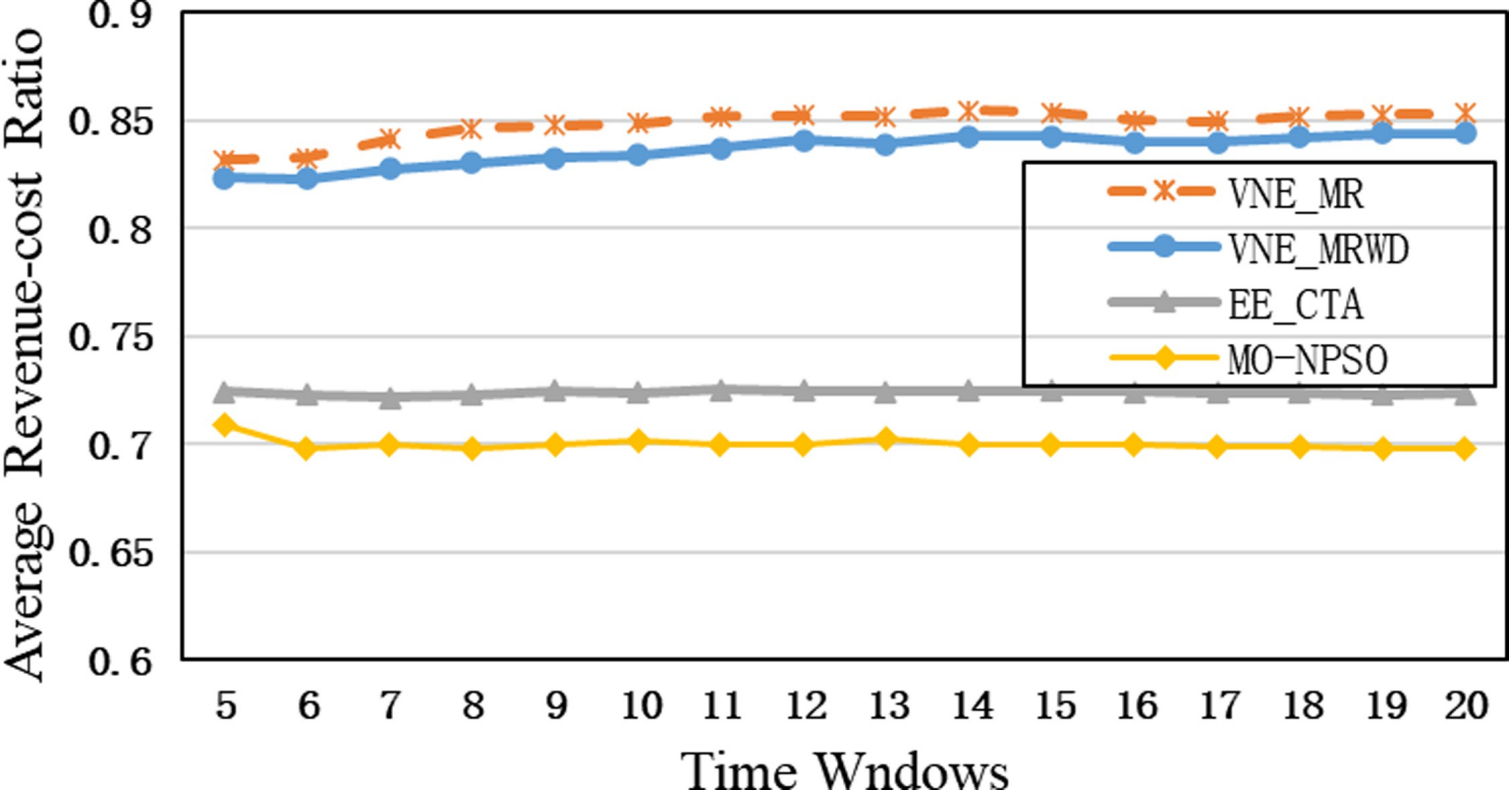
Average revenue-cost ratio.

In the second group, Fig 15 shows that VNE_MR achieves a much lower average computational time compared to EE_CTA. This is because different from the resource availability-based algorithms that require more iterations to obtain node-by-node embedding solutions when exploring the substrate network, VNE_MR operates on a resource management region instead of all nodes in the physical network, and finds the most potential region to host the request by focusing on the graph edit distance between VNR and SN. In addition, the time complexity of our algorithm is *o*(|*N*_*v*_| + |*L*_*v*_| + |*L*_*R*(*Gv*)_| + |*N*_*R*(*Gv*)_|^2^)⋅|*P*^1^|, where $|P^{1}|=\sum_{i=1,2\cdots MG}P_{i}$. It can be seen that the size of our population gradually increases to a scale similar to that of the comparative experiment, which has a direct impact on the computational time. Therefore, as only a small population that is used to search in the SN at the beginning, and they converge to a potential physical area quickly. Only when the regeneration stagnates do we expand the population size by combining the group into a larger population to explore and repeat the previous search phases until we find the near-optimal solution. Furthermore, VNE_MR also achieves this result by pruning the redundant SN information to reduce the size of the search space. Therefore, we only concentrate on the physical resources in *R*(*G*_*v*_), while EE-CTA explores the appropriate physical resources in the SN; obviously, the former’s order of magnitude is much smaller than that of the latter. In particular, although our computational time is sharply reduced, it does not cause the *R*(*G*_*v*_) to skip the global minima in the long run, as the VNE_MR achieved good results in terms of energy savings and revenue-cost ratio compared with EE-CTA. Others, we can also see from Fig 15 that MO-NPSO achieves a computation time that is close to that of VNE-MR, due to the fact that the number of its optimization iterations is similar as ours, but we can also see from Figs 16–21 that MO-NPSO achieves the worst performance in terms of resource management. As shown in Fig 20, the average energy consumption of VNE_MR is reduced by 7,000 W compared with the other two metaheuristic-based VNE algorithms. Fig 21 shows that the revenue-cost ratio of the VNE_MR algorithm is approximately 12% and 15% higher than that of EE_CTA and MO-NPSO, respectively. Figs 16–19 also confirm this result; VNE_MR opens fewer nodes and links and obtains greater utilization than EE_CTA and MO-NPSO. Overall, VNE-MR can guarantee full exploration of the underlying network resources with a small number of iterations, enabling efficient management of resources with a small cost.

Moreover, in Figs 15–21, the yellow dotted line indicates the proposed comparative experiment, VNE_MRWD, without the end-to-end time delay constraint. There is a marginal drop in VNE_MR’s performance across the board compared to that of VNE_MRWD due to the tougher resource requirements. Therefore, the conclusion can be drawn that it is feasible to apply VNE_MR to the VNE problem by simply assigning a new *R*(*G*_*v*_) with personal resource restrictions for virtual networks with different requirements.

## Conclusion

In this study, we used the proposed method to find the near-optimal resource management region in the substrate network to accurately and flexibly control the networked resources in the VNE problem. Two groups of experiments confirmed its resource control ability and flexibility.

In future work, we are planning to conduct further research on network requirements in the contexts of service function chaining or network function virtualization, such as more precise latency and storage, to find a more effective VNE method to control resource allocation accurately when facing personal requirements.

## Supporting information

S1 File Contains all the supporting tables and figures. (ZIP)
